# Identifying cancer cells from calling single-nucleotide variants in scRNA-seq data

**DOI:** 10.1093/bioinformatics/btae512

**Published:** 2024-08-20

**Authors:** Valérie Marot-Lassauzaie, Sergi Beneyto-Calabuig, Benedikt Obermayer, Lars Velten, Dieter Beule, Laleh Haghverdi

**Affiliations:** Max-Delbrück-Center for Molecular Medicine in the Helmholtz Association (MDC), Berlin Institute for Medical Systems Biology (BIMSB), Hannoversche Str. 28, 10115 Berlin, Germany; Charité – Universitätsmedizin Berlin, Corporate Member of Freie Universität Berlin and Humboldt-Universität zu Berlin, Charitéplatz 1, 10117 Berlin, Germany; Centre for Genomic Regulation (CRG), The Barcelona Institute of Science and Technology, 08003 Barcelona, Spain; Universitat Pompeu Fabra (UPF), Barcelona, Spain; Core Unit Bioinformatics, Berlin Institute of Health at Charité – Universitätsmedizin Berlin, 10117 Berlin, Germany; Centre for Genomic Regulation (CRG), The Barcelona Institute of Science and Technology, 08003 Barcelona, Spain; Universitat Pompeu Fabra (UPF), Barcelona, Spain; Core Unit Bioinformatics, Berlin Institute of Health at Charité – Universitätsmedizin Berlin, 10117 Berlin, Germany; Max-Delbrück-Center for Molecular Medicine in the Helmholtz Association (MDC), Robert-Rössle-Str. 10, 13125 Berlin, Germany; Max-Delbrück-Center for Molecular Medicine in the Helmholtz Association (MDC), Berlin Institute for Medical Systems Biology (BIMSB), Hannoversche Str. 28, 10115 Berlin, Germany

## Abstract

**Motivation:**

Single-cell RNA sequencing (scRNA-seq) data are widely used to study cancer cell states and their heterogeneity. However, the tumour microenvironment is usually a mixture of healthy and cancerous cells and it can be difficult to fully separate these two populations based on transcriptomics alone. If available, somatic single-nucleotide variants (SNVs) observed in the scRNA-seq data could be used to identify the cancer population and match that information with the single cells’ expression profile. However, calling somatic SNVs in scRNA-seq data is a challenging task, as most variants seen in the short-read data are not somatic, but can instead be germline variants, RNA edits or transcription, sequencing, or processing errors. In addition, only variants present in actively transcribed regions for each individual cell will be seen in the data.

**Results:**

To address these challenges, we develop CCLONE (Cancer Cell Labelling On Noisy Expression), an interpretable tool adapted to handle the uncertainty and sparsity of SNVs called from scRNA-seq data. CCLONE jointly identifies cancer clonal populations, and their associated variants. We apply CCLONE on two acute myeloid leukaemia datasets and one lung adenocarcinoma dataset and show that CCLONE captures both genetic clones and somatic events for multiple patients. These results show how CCLONE can be used to gather insight into the course of the disease and the origin of cancer cells in scRNA-seq data.

**Availability and implementation:**

Source code is available at github.com/HaghverdiLab/CCLONE.

## 1 Introduction

Cancer is a multistep process driven by somatic mutations in which healthy cells progressively evolve into cancerous states. Quantifying how cancer cell states differ from healthy states helps us understand this disease and provides potential therapeutic targets. Single-cell RNA sequencing (scRNA-seq) has emerged as a powerful tool to study cancer cell states and their heterogeneity. However, the sampled tumour microenvironment is usually a mixture of healthy and cancerous cells (Hanahan and Weinberg 2011), and fully separating these two populations can be difficult based on transcriptomics alone. Measuring mutational status and gene expression in the same single cell would allow us to more accurately identify the cancer population through mutations and relate that information to the observed transcriptional states. However, sequencing the entire genome and transcriptome of the same individual cell is costly and has low throughput (Dey *et al.* 2015, Macaulay *et al.* 2015, Cheow *et al.* 2016). Another option is to sequence only targeted genetic regions containing somatic mutations alongside the transcriptome (Nam *et al.* 2019, Petti *et al.* 2019, Rodriguez-Meira *et al.* 2019, Van Egeren *et al.* 2021), but this requires prior knowledge on the position of these mutations in each sample. These positions are usually inferred from preceding bulk DNA sequencing or through panel testing of known cancer-associated genes and carry additional costs and delays.

Computational methods allowing us to estimate the mutational status directly from the data provided by a standard scRNA-seq experiment would provide a cost-efficient solution to this problem. In the past, large copy number variants (CNVs) have been used to identify cancer populations in scRNA-seq data (Fan *et al.* 2018, Gao *et al.* 2021, 2023). As the CNVs cover large areas of the genome, the tools leverage the read data across multiple adjacent regions to identify these CNVs. Mitochondrial variants (MVs) with high heteroplasmy have also proven helpful to study the cell lineage in cancer and healthy tissue due to the high mutation rate of the mitochondrial genome, large number of mitochondria per cell, and strong expression of mitochondrial genes (Ludwig *et al.* 2019, Velten *et al.* 2021, Kwok *et al.* 2022). However, this approach is limited to the subset of cancers and samples that have CNVs or high heteroplasmy MVs.

Single nucleotide variants (SNVs) observed in scRNA-seq data could also identify cancer cells. However, calling somatic SNVs confidently from scRNA-seq data is a challenging task. First, only variants present in actively transcribed regions will be seen in the data. Even then, we might not catch these variants in every cell due to the low average coverage of individual positions and of allelic dropout. Furthermore, when calling variants against the human reference genome, most variants seen in scRNA-seq reads are not somatic mutations but can be germline variants, RNA edits or transcription, sequencing, or processing errors. In other words, we will completely or partially miss most somatic SNVs, and most identified SNVs will not be somatic.

Despite these challenges, recent studies suggest that SNVs called from scRNA-seq data can be used to identify cancer cells. Usually, all observed SNVs are filtered to retain only SNVs that are expected to be somatic. This is done either by trying to identify the cancer driver variants (Yizhak *et al.* 2019, Gasper *et al.* 2022), by using cell type information to find variants that are unique to each lineage (Muyas *et al.* 2024), or by leveraging allele co-segregation patterns (Dou *et al.* 2024). Nevertheless, there is still some uncertainty in the prediction of SNVs as somatic and not all cells or lineages may contain well covered high-confidence predicted somatic SNVs. Furthermore, the existing studies do not directly offer a computational framework to link the potential somatic SNVs to cell clonal assignments. We hypothesized that by using a model which accounts for the uncertainty in the data, and leverages information across multiple co-occurring variants we could incorporate more variants (including low confidence and potential passenger variants) for identification of the cancer cell lineage.

We note that the sampled populations potentially consist of a mixture of subpopulations each characterized by their own unique mutations and sharing some founding mutations. If we were capturing all these events, we could fully reconstruct this hierarchy. However, as we only observe a small subset of somatic variants, we will only be able to identify large enough (sub-)populations characterized by these variants. In this work, we use the term ‘clone’ to define a big population of cells that share several somatic variants. Because cancer cells grow fast and acquire multiple somatic mutations, the clones we identify most often coincide with cancer clones. However, depending on when these variants were acquired in the cancer evolutionary tree, they might identify all cancer cells (and thus potentially a mixture of multiple subpopulations) or only a subset of the cancer cells.

In this work, we show that SNVs called directly from scRNA-seq data can be used to identify cancer clones. We introduce CCLONE (Cancer Cell Labelling On Noisy Expression), a tool adapted to handle the uncertainty in this big data. CCLONE is a fully automated tool that can be applied on new or existing scRNA-seq samples. We validate our results on three single-cell datasets with known cancer cell identities inferred based on targeted amplification of known SNVs, MVs, and CNVs. The first two datasets present 19 patients with acute myeloid leukaemia (AML) (Velten *et al.* 2021, Beneyto-Calabuig *et al.* 2023), which is a cancer typically characterized by a low mutation load (Ley *et al.* 2013). The third dataset presents seven lung adenocarcinoma patients (Bischoff *et al.* 2021) which is typically characterized by a much higher mutational load (Ellrott *et al.* 2018). For multiple patients, our method is able to identify the known clones without using any prior information on the samples. The method also returns a set of SNVs enriched in each identified clone along with their expected allele frequency (i.e. homo/hetero-zygocity). We show that these variants enhance interpretability of the results and points to real somatic events which inform about the course of disease evolution and the origin of cancer cells.

## 2 Materials and methods

### 2.1 Variant calling and filtering

#### 2.1.1 Preparing the raw data and variant calling

The raw data were aligned to reference genome hg38 (Lander *et al.* 2001) with Star version 2.7.8a (Dobin *et al.* 2013) for the MutaSeq data and Cell Ranger version 7.1.0 for the three 10X datasets. Variants were called on single cells with Cellsnp-lite version 1.2.3 (Huang and Huang 2021). We annotate the variants with VEP (McLaren *et al.* 2016) with custom annotation of common dbSNP germline variants (MAF ≥0.01 in at least one major population) (Sherry *et al.* 2001) and RNA edits found in REDIdb (Picardi *et al.* 2007).

#### 2.1.2 Variant filtering

To minimize potential artefacts in variant calling, we filter all variants found in repeat regions, according to RepeatMasker (Smit *et al.* 1996–2010). We filter all variants annotated as RNA edits. We remove all low coverage variants found with cov ≥2 in <10% of the cells.

To allow flexibility in filtering of germline variants and low coverage variants, we create six different variant subsets corresponding to the combination of different thresholds. These thresholds are exclusion/inclusion of germline variants and exclusion of variants with MAF of 2%, 5%, or 10%. We then run the wNMF on each subset and later choose the most informative subset.

### 2.2 Clustering of cells and variants using wNMF

#### 2.2.1 Input

The wNMF takes as input the variant observation matrix *M* and the confidence (weight) matrix *W* (both of dimension *n_cells_*, *n_vars_*). *M* is chosen to represent our best estimation of the true VAF, while *W* reflects the confidence we have in each value of *M*.

When working with nuclear variants, *M* contains the discretized (into three values 0, 0.5, and 1 corresponding to homozygous reference, heterozygous, and homozygous variant observations) VAF for every variant in every cells. We count an allele as observed, if we see at least one UMIs (or 1 reads for non-UMI technology) matching that allele, and an allelic frequency ≥0.05. We have:

(1) $$M_{ij}=\{\begin{array}{cc} 1 & \text{if we see only ALT reads}\\ 0.5 & \text{if we see both REF and ALT reads}\\ 0 & \text{if we see only REF reads}\\ 0 & \text{if we see none of REForALT}(i.e.\;\text{not expressed}) \end{array}$$

The *W* matrix is then chosen to reflect the confidence we have in each value in *M*. We define *W_ij_*, the probability for the observation *M_ij_* by:

(2) $$W_{ij}=\{\begin{array}{cc} 0.5 & \text{if we see only ALT reads}\\ 1 & \text{if we see both REF and ALT reads}\\ 0.5 & \text{if we see only REF reads}\\ 0 & \text{if we see none of REF or ALT}\; \end{array}$$

#### 2.2.2 wNMF

In NMF, we factorize the observation matrix *M*, into two matrices *C* of size (*n_obs_*, *K*), and *V* of size (*K*, *n_vars_*), with the constraint that these matrices have no negative elements. In wNMF, we further weight each value in *M* by its weight defined in *W* according to Equation (4). We note that with the weights defined in Equation (2), positions that are not covered will not contribute to the cost.

Since the solution to Equation (4) is not unique, we find the optimal *C* and *V* matrices via an EM procedure; first *C* is (randomly) assigned and *V* is found by a nonnegative least squares solver. Then *V* is fixed and *C* is solved analogously. We iterate these two steps until convergence (default of 100 EM iterations, chosen based on Supplementary Fig. S20). Supplementary Figure S20 demonstrates the robustness of the final solution with respect to the initial (random) initialization on real data.

Ideally, the number of latent factors *K* would reflect the number of co-occurring variant groups clearly identifiable from the data. If this information is known, the corresponding *K* can be used as input to the wNMF. In the absence of prior knowledge, we try to determine the best number of factors as defined bellow.

### 2.3 Evaluation and selection of results

#### 2.3.1 Bootstrapping

The method to fit our wNMF does not guarantee finding a global minimum of the cost function, and the final factor matrices can vary over multiple rounds with random initialization. To test its robustness with respect to noise in input data, we bootstrap the wNMF by randomly subsampling 90% of the variants and recomputing the factor matrices, with default of 50 bootstrap (choice of this default shown in Supplementary Fig. S20). We then align the results and get the mean and variance of the 50 bootstrap matrices. The mean *C* and *V* matrices gives us the final assignments, while the variance of the bootstrap matrices indicates how robust these assignments are over multiple bootstrap iterations.

#### 2.3.2 Selection of result

We run our wNMF on the different variant subsets and different number of factors *K*, and select the best output as final result. Because the variant subsets include different number of variants which can have different properties (different types of event included, different MAF), the weighted sum of squared errors E is not directly comparable. Instead, we compare the cell factors *C* that have the same dimension for all subsets. We expect the cell factors to reflect our genetic clones, and for each cell to be predominantly assigned to one clone. Hence, the best result is chosen as the one with the largest (i.e. closest to 0 for negative values) orthogonality score *s* between the clones:

(3) $$s=-\frac{1}{\left(\begin{array}{c} K\\ 2 \end{array}\right)}\sum_{i\neq j}^{K}\frac{C_{.i}\cdot C_{.j}}{\left||C_{.i}||\;||C_{.j}|\right|}$$

where $C_{.i}$ and $C_{.}j$ indicate the *i*th and *j*th columns of *C* respectively, and the nominator presents the inner product between them. The denominator presents the norm of the vectors.

Supplementary Figure S21 demonstrates the effectiveness of this approach on real data.

#### 2.3.3 Factor annotation and validation

After selection of the best subset, we get the cell factor weights *C* and the variant factor weights *V*. If the method succeeds we expect the factors to reflect genetic clones, i.e. one or multiple factors for healthy clone(s) and one or multiple factors for cancer clone(s). However, the wNMF does not directly label these factors as healthy or cancer. In this work, we label the factors based on prior knowledge of cancer or healthy cell types. Cell type annotation was taken from the original studies for all datasets. Factors containing no cells of a known healthy cell type labelled from expression (s.a. T-cells in AML) are labelled as cancer and the others as healthy. In the absence of sufficient known healthy cell from expression we label the factors analogously based on cells of a known cancer cell type labelled from expression (s.a blasts in AML).

If all factors contain cells of a known healthy cell type (or cancer cell type), we consider the model to have failed. More details in Supplementary Note SC and alternatives are discussed in Supplementary Note SD.

## 3 Results

In this section, we first introduce and describe the CCLONE workflow. We then validate the tool on the AML and lung adenocarcinoma datasets, and show examples where the method helps get a better understanding of the analysed samples. Lastly, we show how the method’s likelihood of success depends on the data quality (especially sequencing coverage) and the resulting capture of sufficient somatic variants.

### 3.1 CCLONE workflow

For a fixed set of run parameters, the CCLONE workflow includes three main steps; (i) Variant calling and filtering, (ii) Clustering cells and variants using wNMF, (iii) Evaluation and selection of result. CCLONE runs this workflow for multiple sets of parameters and returns the clearest result as the final output.

**Figure 1.**
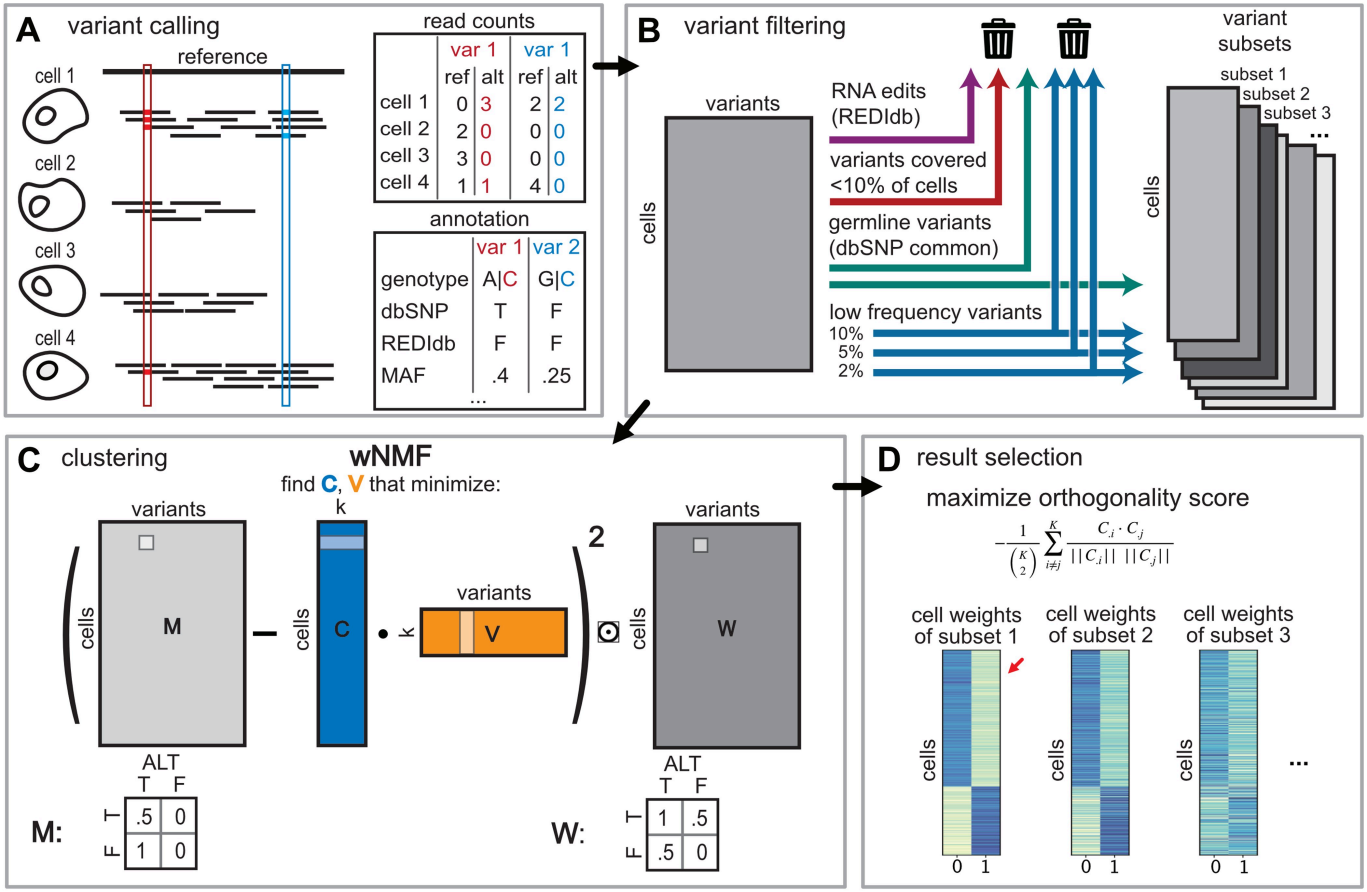
CCLONE workflow. (A) Variants are called from single-cell short-read data. The short reads differ from the reference in two positions (outlined in red on the left and blue on the right). For every variant, we keep the number of alternative and reference counts, and extract variant annotation. (B) We filter the variants based on database annotation as well as coverage and frequency. Because the best filtering threshold might differ between samples, we try different thresholds resulting in multiple variant subsets. (C) We use a weighted NMF to discover the hidden clonal structure in the variant call data. For this, the read count data are transformed into the observation matrix M corresponding to the discretized VAF, and a weight matrix W that reflects the confidence that we have in each value in M. The wNMF is calculated for every variant subset and a range of number of factors *K*. (D) To select the best result, we compare the computed cell factors (matrix C) for every subset and *K*, and select the wNMF output with the largest (i.e. closest to 0 for negative values) orthogonality score *s* between the factors [Equation (4)] as this reflects a clearer separation between the clones.

As input, CCLONE takes annotated variant call data from scRNA-seq data (Fig. 1A). In this work, we use variants called from Cellsnp-lite (Huang and Huang 2021) against the human reference genome. CCLONE then filters the variants to get rid of the bulk of spurious variants most of which are nonsomatic (Fig. 1B). RNA edits, whose occurrence patterns are highly cell type specific (Cuddleston *et al.* 2022), and thus a potential confounder in the data, are filtered out based on reference annotation in REDIdb (Picardi *et al.* 2007). Variants with very low coverage, or very low minor allele frequency (MAF) cannot reliably be leveraged to identify genetic clones and are also filtered out. Heterozygous germline variants are expected to be found equally in all cells. However, their occurrence can be associated to somatic events if they are located within regions either lost through a deletion or loss of heterozygosity event (LOH), or not expressed through strong imbalance in allelic expression (potentially due to a somatic variant in the regulatory region). Because of high variability between cancers, types of mutations and clone population size, a different filtering threshold might produce best results for different samples. To allow the model to make use of as many somatic variants as possible, while excluding as many nonsomatic variants as possible, we try different filtering thresholds and later [in step (iii)] allow the model to select the most informative set. We note that our filtering is very lenient to try to include and use uncertain variants, and even after filtering most variants in the data are still likely not somatic. Because of this, simply looking at the variant load in individual cells is not sufficient to label them (Supplementary Figs S1 and S2), and we need a method adapted to deal with this data.

After filtering, we want to recover the hidden clonal structure in the cell-variant call matrix *M*. We expect somatic variants in the input matrix *M* to co-occur within genetic clones. However, this co-occurrence signal is hidden in the noisy (through the presence of nonsomatic variants) and sparse input data. Nonnegative matrix factorization (NMF) has been widely used to capture hidden structure in scRNA-seq data (Zhu *et al.* 2017, Wu *et al.* 2020) because of its capacity to deal with random noise. However, NMF assumes that the data are complete, while we will only observe a variant if the position is covered, i.e. actively expressed in that cell. To account for this additional uncertainty associated with coverage, in step (ii), we use a weighted version of NMF (wNMF), that uses a weight matrix *W* to reflect how confident we are in each value of the variants call matrix *M* (Fig. 1C) based on each variants’ coverage in each cell. In particular missing values are given a weight of 0, so that they do not contribute to the error. We discuss the choice of the weights in Supplementary Note SA and Supplementary Figs S3 and S4.

(4) $$E=\sum_{i=1}^{n_{\mathrm{cells}}}\sum_{j=1}^{n_{\mathrm{vars}}}{\left(\sum_{k=1}^{K}M_{ij}-C_{ik}V_{kj}\right)}^{2}W_{ij}$$

We run the wNMF on all variant subsets and a range of number of factors *K*, and in step (iii) chose the best result. To select the best result, we use the assumption that if the clones are clearly distinguishable in the variant data and captured by the model, then the cell factors reflecting these clones should be clearly separated, i.e. uncorrelated. We select the best result based on the orthogonality score *s* of the cell factors [Fig. 1D, Equation (3)]. Alternatives are discussed in Supplementary Note SB. *s* as a function of *K* and the subset is shown in Supplementary Fig. S5 for all patients analysed in this work.

After running CCLONE, the cell factors give us the clonal assignments while the variant factors reflect the variants enriched in these clones and can be used to find disease relevant somatic events. The factors are not directly labelled as healthy or cancer, but can be labelled through prior knowledge. In this work, we use the cell type annotation based on expression extracted from the original studies for that effect (see Methods and Supplementary Note SC). We consider the model to be successful if we find both healthy and cancer factors. This ensures that the captured factors correspond to the known lineages, and that the separation between the two is well defined in the data. Alternatives are discussed in Supplementary Note SD.

### 3.2 Application on AML patients datasets

We validate CCLONE on two AML single-cell datasets. AML is usually characterized by a low mutation load. These mutations cause a block in differentiation of the haematopoietic stem cells (HSCs) resulting in the malignant expansion of aberrant progenitor cells called ‘blasts’ (Zeng *et al.* 2022). This population is fuelled by leukaemic stem cells (LSCs) that are transcriptionally similar to normal HSCs and difficult to target. Because of the low mutation load and the presence of difficult to identify LSCs, AML provides a good test case on which to validate our method. The first analysed dataset contains four patients sequenced with SmartSeq2 (Velten *et al.* 2021), and the second dataset contains 15 patients sequenced with 10X (Beneyto-Calabuig *et al.* 2023) (Supplementary Fig. S6 shows the cell types and patient labels on UMAP). In both of these datasets, the cell labels as healthy or cancer could previously be recovered in some patients (respectively 2 and 11 patients) based on MVs and targeted amplification of known somatic SNVs in Velten *et al.* (2021) and additionally through CNV in (Beneyto-Calabuig *et al.* 2023). The two additional Smart-Seq2 patients had partial cell labels based on a single nuclear somatic variant each. We call these previously recovered labels ‘reference labels’ in the following sections.

#### 3.2.1 CCLONE identifies cancer cells

**Figure 2.**
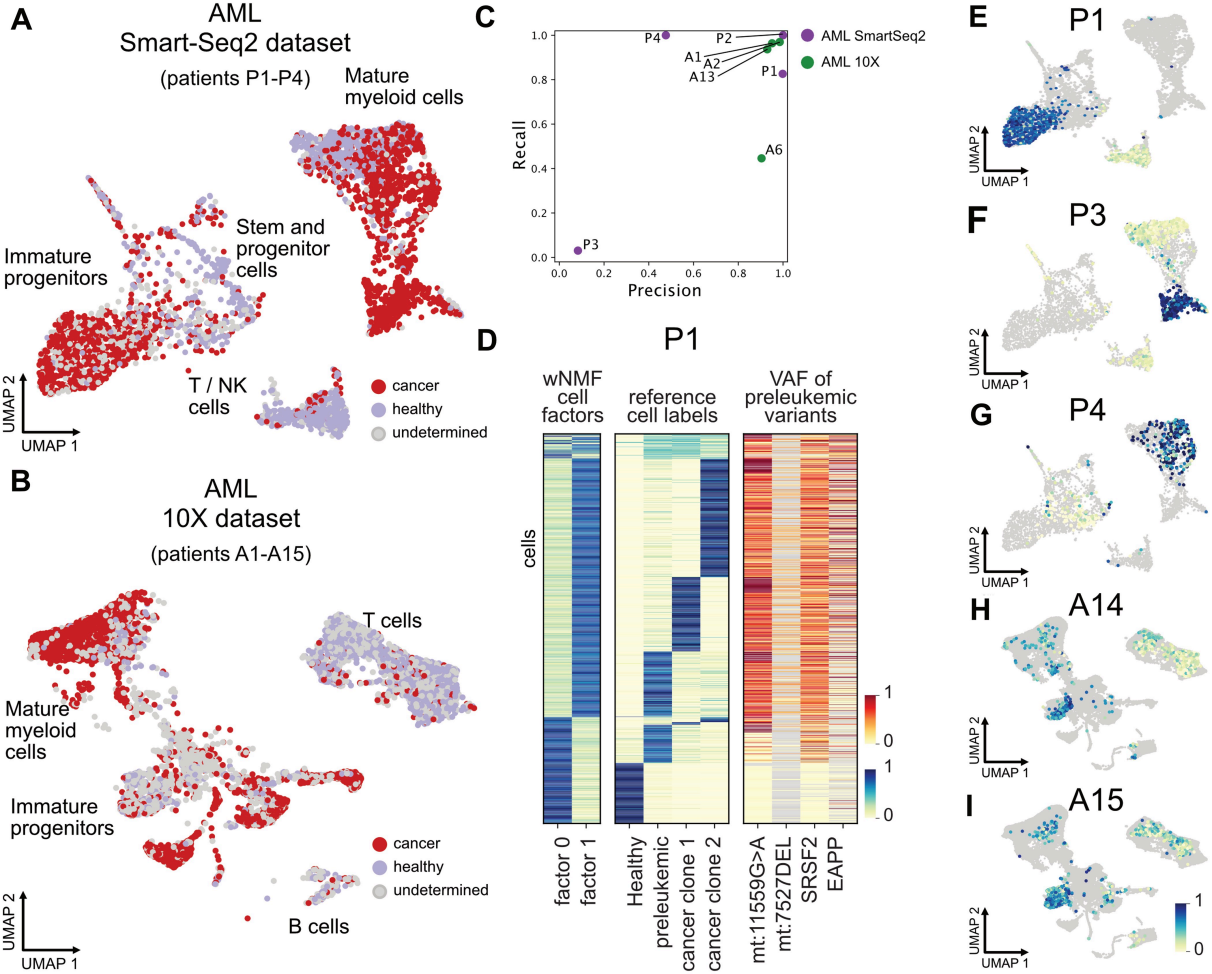
Cell factors capture genetic clones. (A and B) Cell assignments to healthy or cancer based on the wNMF cell factors plotted on the UMAP for the AML Smart-Seq2 dataset (Velten *et al.* 2021) in A and the AML 10X dataset (Beneyto-Calabuig *et al.* 2023) in B. (C) When present, we compare our cancer cell labels to the reference cancer cell labels from each dataset, and return the precision and recall for every patient. (D) Heatmap comparing the wNMF cell factors to the reference cell labels and to the VAF of the variants used to separate the preleukaemic population from the healthy one. (E–I) Cancer cell factors for patient P1, P3, P4, A14, and A15 coloured on the UMAP. Cells from the other patients of the dataset are shown in grey for ease of comparison.

CCLONE successfully identified cancer clones in all four SmartSeq2 patients (patients P1–P4 in Fig. 2A) and six of the 10X patients (patients A1, A2, A6, and A13–A15 in Fig. 2B). We use the T cells and Blasts to label the factors (Supplementary Note SC). Cells with similar weights for the healthy and cancer factors (difference in weights < 0.3) are labelled as undetermined. The method clearly groups the aberrant progenitors and the T cells in separate factors, while the stem cell populations are a mixture of healthy and cancer cells. We compare the labels for the patients where we have both reference cell labels and new labels (Fig. 2C) and find a near complete agreement for patients A1, A2, A13 and P2.

For patient P1 and A6, we are identifying a subset of the cells labelled as cancer in the reference. For these patients, it is possible that our method identifies a subclone of the reference, as shown in the example of patient P1 (Fig. 2D and E). Here, the reference differentiates healthy cells, and the cancer population containing the preleukaemic clone and two cancer subclones. Comparing the wNMF cell factors to the reference labels, we see that the reference healthy cells are predominantly assigned to factor 1, while the two cancer clones are assigned to factor 0. The preleukaemic population is split between the two factors. The preleukaemic cells assigned to factor 1 have much lower variant allele frequency (VAF) of the two MVs mt:7527DEL and mt:1159G>A than the other preleukaemic and leukaemic cells. This indicates that the preleukaemic population could potentially be further separated in two subclones as separated by our factors.

For patient P3 and P4 the reference is based on a single low-coverage nuclear variant each. For P3 there is no agreement between the reference and new labels. The reference is based on a single IDH2 variant that is depleted in the cells that we identify as cancer (Fig. 2F and Supplementary Fig. S7). The IDH2 mutated cells are transcriptionally similar to monocytes, which could be either healthy or cancer. However, the population that we identify as cancer is transcriptionally very aberrant (e.g. expressing HBZ), indicating that they must be cancer cells and were missed previously. This points towards there being two distinct genetic clones, each of which is captured by one approach. For patient P4 (Fig. 2G), the reference is based on a single very low coverage variant, and likely missed in many cancer cells (Supplementary Fig. S8). Here, CCLONE provides more complete cancer cell labels and potentially now captures all cancer cells instead of only a subset.

For patients A14 and A15 we have no reference cell labels, but CCLONE could still identify a cancer population based on SNVs (Fig. 2H and I). These patients had no well-covered known leukaemic SNVs and additionally no usable CNVs or MVs. This highlights the advantages of using a method that does not rely on prior knowledge of existing somatic SNVs, and consequently is not restricted to a small subset of the observed SNVs. Patients P3 and P4 are other examples where previous methods based on MVs and known leukaemic SNVs are not sufficient to fully label the cancer cells. We further validate the clones based on known cancer and healthy cells within clones (Supplementary Fig. S9).

In this work, we do not use the MVs, nor directly call the CNVs for use with CCLONE. This means that we are potentially using a different set of somatic events than the reference to characterize the cancer clone. This can explain why we do not find the same exact same clones as the reference if the different variant subsets are found in different subclones.

#### 3.2.2 CCLONE finds cancer-associated variants

On top of the cell factors, the wNMF also returns variant factors. To find clone-associated variants, we extract the variants with the largest difference in weight between the factors (>0.3) and covered in at least 20% of the cells assigned to each clone. For the AML Smart-Seq2 patients, we have a whole exome (WE) cancer and control sample which we can use to validate and understand the variant factors.

**Figure 3.**
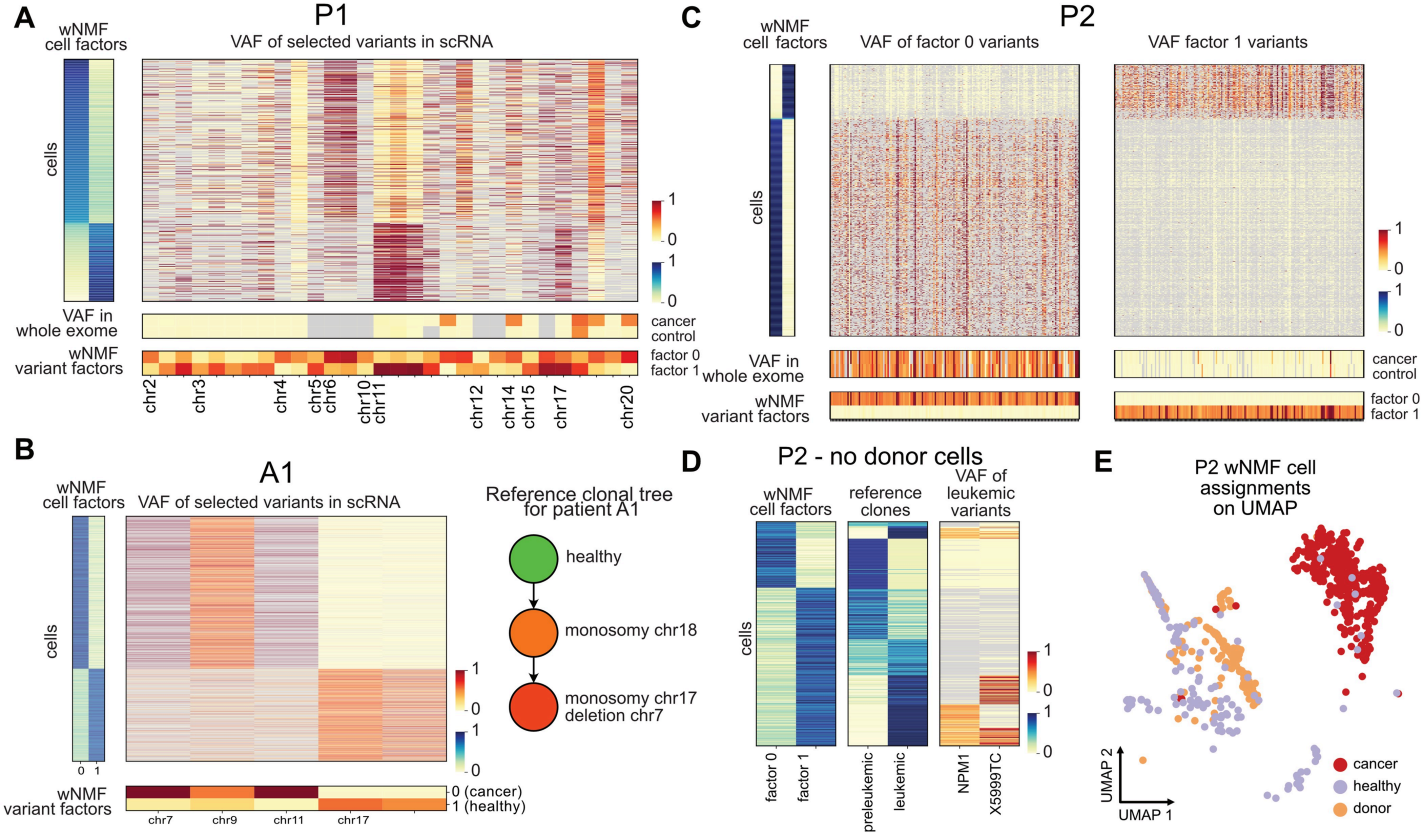
Variant factors capture somatic mutation events. (A) VAF for selected variants for the cells of patient P1. The cells are sorted by cell factors and the subset of variants are selected based on difference of weight between the variant factors (>0.3). Grey values have too low coverage (≤2 reads for scRNA and ≤5 for whole exome data). (B) VAF of selected variants (difference of weight >0.3) for patient A1. The reference clonal tree was extracted from (Beneyto-Calabuig *et al.* 2023), and is based on their method CloneTracer. (C) VAF of selected variants (difference of weight >0.3) for patient P2. The enriched variants in each factor are shown on two separate heatmaps for ease of visualization of the different frequencies of observing these variants in the WE samples. Factor 1 (variants shown on right heatmap) corresponds to donor cells, and the variants characterizing these cells are almost never found in the whole exome cancer or control, compared to the variants found in factor 0. (D) After excluding the donor cells and recalculating the wNMF, we compare the wNMF cell factors to the reference cell labels and to the VAF of the variants used to identify the leukaemic population for P2. (E) Final CCLONE cell assignments for P2.

The clone-associated variants of patient P1 are shown in Fig. 3A. Four of the SNVs associated with the cancer factor are supported by the WE, indicating that CCLONE is capturing somatic events. For P1 these four variants are also the only variants enriched in the WE cancer (Supplementary Fig. S10) and with sufficient coverage in the single-cell data. A lot of the variants with high difference in weight between the clones are not found in WE, indicating that these could either correspond to variants characterizing small subclones or some other genomic signal manifested in the noisy and imperfectly filtered variant calls. The VAFs of these variants show a very clear difference between the clones, and they co-occur with the known somatic SNVs, thus they can also be used by the wNMF to identify the clones. Patient P4 shows similar patterns to patient P1 (Supplementary Fig. S8).

For some patients, we observe that variants observed at VAF close to 0.5 in the healthy populations are either lost (VAF≈0) or fixated (VAF≈1) in the cancer clone (Patient A1 in Fig. 3B, P3 in Supplementary Fig. S7 and A2 in Supplementary Fig. S11). In these cases the method could be capturing CNV deletions or LOH, resulting in the loss of one allele and the heterozygosity of the germline SNVs overlapping that region. Figure 3B shows the example of patient A1, where the monosomy on chromosome 17 was also captured by the wNMF. The other events on chromosome 7 and 11 point to further losses in those regions, one of which could correspond to the known deletion on chromosome 7. Another example is given patient P3 (Supplementary Fig. S7), where the variants separating the cancer clone from the other cells are predominantly germline variants (found in both WE cancer and control), and variants heterozygous in the healthy population are either lost of fixated in the subclone.

For patient P2 (Fig. 3C), we find a very high number of variants associated with each clone, and those associated with clone 1 are found in both WE samples while those associated with clone 0 are found in none. Both of these variants subsets are enriched in known germline SNVs (dbSNP common). The extremely high number of likely germline variants clearly separating both factors point towards this patient having cellular mosaicism. Such blood microchimerism can arise naturally if the patient had a twin through exchange of haematopoietic stem cells in utero, or after pregnancy (Drexler and Wagner 2006, Shrivastava *et al.* 2019). This was overlooked in previous analysis, and the reference cell labels reflect the separation between patient (factor 0) and ‘donor’ cells (factor 1). Here, considering both cells and variants jointly allows us to get a more complete picture of the data. Excluding the donor cells, and rerunning variant filtering and wNMF, we find two clones, corresponding in parts to the separation between preleukaemic and leukaemic in the original publication (Velten *et al.* 2021) (Fig. 3D and E). The main disagreement between the reference labels and our labels are in cells with low or no coverage for the variants used to label the reference (Fig. 3D), indicating that CCLONE is helping us refine the labels and now identifies all leukaemic cells. This separation between healthy and cancer for P2 could only be identified after excluding the donor cells and the associate variant, as the signal-to-noise ratio for this pattern was otherwise too low. This highlights the importance of variant set selection.

The VAF plots of selected variants for all additional AML 10X patients are shown in Supplementary Fig. S11, and for patients P2 excluding donor cells in Supplementary Fig. S12.

### 3.3 Data quality and number of captured variants determine success

**Figure 4.**
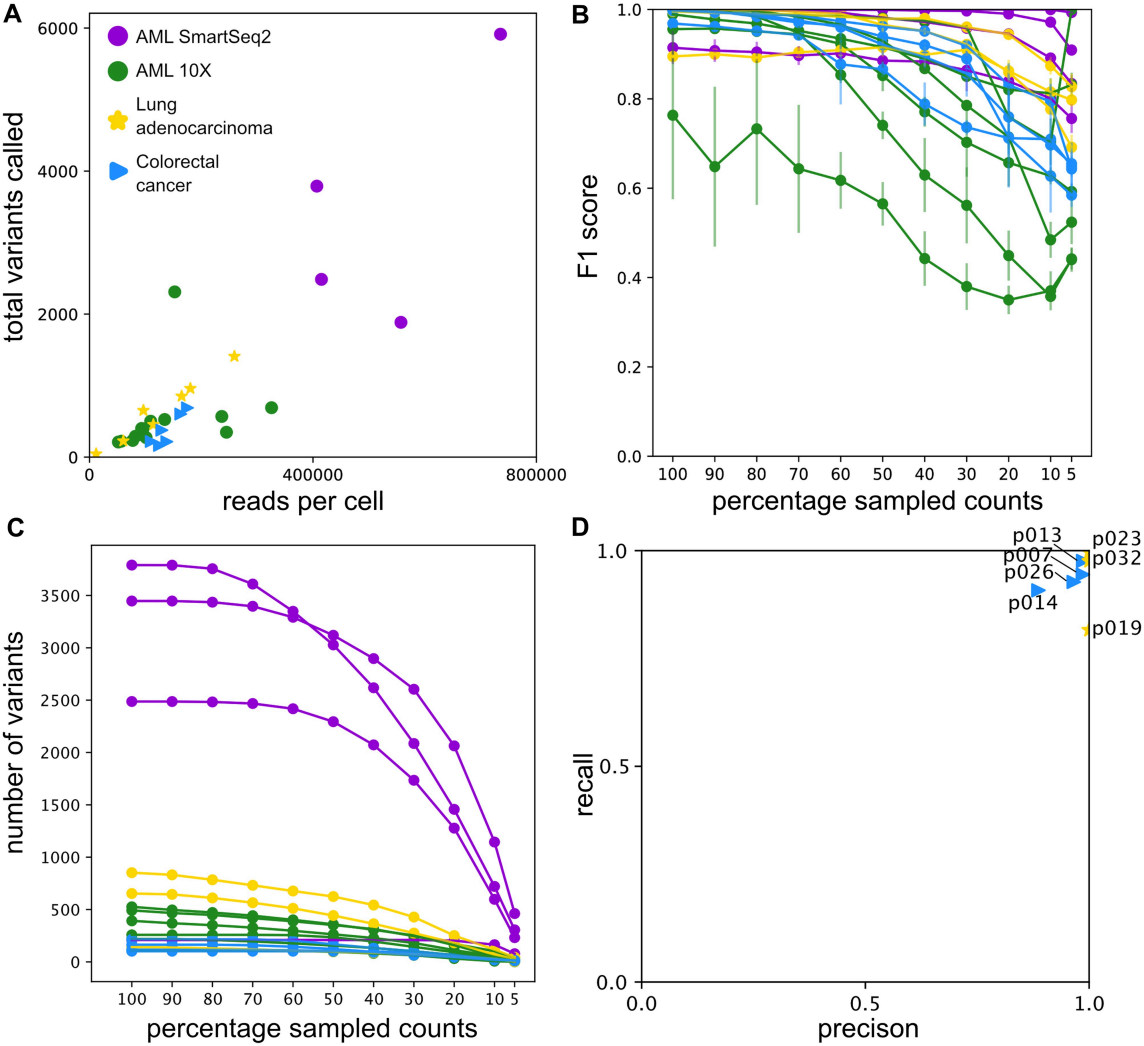
Method success is dependent on the quality of the data. (A) Total number of called variants as a function of the number of reads per cell for each patient. Here we include all called variants covered in 10% or more of the cells and observed in >2% of covered cells. (B–C) For every patient we randomly subsample counts from the reference and alternative count matrices, and rerun the wNMF on the subsets. Comparing the cancer cell labels between the full dataset and the subset, we get the F1 score (in B) and the number of variants with sufficient coverage (in C) as a function of the percentage of sampled counts. (D) We compare our cancer labels with the reference cancer label and report the precision and recall for the lung adenocarcinoma (shown as yellow stars) and CRC patients (shown as blue triangles).

CCLONE does not succeed in recovering clonal structure for all AML patients. The model needs multiple co-occurring somatic events to identify the clones, and these might not be found at sufficient frequency for all the patients. In particular, the rate of success for the AML Smart-Seq2 data (4/4) is higher than for the 10X ones (6/15). This is likely due to both higher sequencing depth of individual positions and sequencing breadth across positions (as Smart-Seq2 is not 3′ biased unless 10X), resulting in a much higher number of variants captured for Smart-Seq2 than for 10X (Fig. 4A). To simulate the success rate at lower sequencing depth, we subsample counts from our input data and rerun CCLONE. We then compare the performance of the subsampled data to the full data in Fig. 4B. As expected the precision significantly declines at lower simulated sequencing depths, although with high variability between patients, reflecting the variation in clarity of the signal. Interestingly, the performance for Smart-Seq2 stays relatively high even at lower subsampled coverage percentages. This is likely due to the fact that we still capture significantly more variants in Smart-Seq2 than 10X (Fig. 4C), even at low subsampled coverage percentages, likely due to the higher breadth of coverage of Smart-Seq2.

As the success of the wNMF relates with the number of captured somatic events, we hypothesized that for tumours with higher mutation load we might still succeed in capturing the clones at low coverage. To test this, we applied CCLONE on a 10X lung adenocarcinoma (Bischoff *et al.* 2021) with seven patients, shown in yellow in Fig. 4. We also tested the method on a colorectal cancer (CRC) (Uhlitz *et al.* 2021, Wei *et al.* 2024) dataset with six patients shown in blue in Fig. 4. Preprocessing of these datasets is detailed in Supplementary Note SE and Supplementary Fig. S13. Patient and cell type labels are shown in Supplementary Figs S14 and S15.

CCLONE succeeds for 7 of these 13 patients (Fig. 4D and Supplementary Fig. S16) and the VAF plots of selected variants (Supplementary Fig. S17) help us identify likely somatic events. In the selected variants we see both patterns that resemble CNV losses or LOH (s.a. the ones seen in A1), and also patterns that point towards acquisition of somatic nuclear variants. Nevertheless, the overall success rate for the lung dataset is lower than our initial expectation when compared with the AML datasets. This result highlights that coverage is a more important determinant of CCLONE’s success rate rather than the mutation load, as this considerably increases the chance of capturing sufficient co-occurring somatic variants.

Another determinant of success is the presence of clonal populations of sufficient size in the data. If the clonal population size is too small, the patterns of co-occurrence will approach background noise and cannot be identified. Because of this we exclude samples containing <3% of known healthy cells (based on cell types) in this analysis (Supplementary Note SE and Supplementary Fig. S13).

### 3.4 Computational efficiency

The two most computational expensive steps of CCLONE are variant calling and wNMF. We report the runtime on a Dual Xeon E5-2650v2 (8cores/2.6 GHz) and 15 GB of memory for variant calling with Cellsnp-lite and for the wNMF in Supplementary Fig. S18. The runtime scales linearly with the input size and the number of iterations.

For a 10X patient with 10000 cells, variant calling with Cellsnp-lite over all chromosomes in parallel, we estimate to take about 48 h. Depending on the number of variants, subsequent analysis of the variant calls with CCLONE we estimate to take up to 12 h.

## 4 Discussion

In this manuscript, we show that cancer clones can be identified from SNVs called directly from scRNA-seq data. These calls tend to contain many nonsomatic variants and often have very low to no coverage in individual cells. We introduce CCLONE, a method adapted to work with uncertain variant calls. We validated the method on two AML datasets a lung adenocarcinoma dataset and a CRC dataset, and show that the method captures genetic clones. The interpretable output of the method also allows us to find disease associated variants pointing to somatic events. By jointly considering all cells and all variants to find patterns of co-occurrence, CCLONE can identify clonal patterns from scRNA-seq data. CCLONE thus allows the multi-modal analysis of single cells in scRNA-seq data by studying genetic information in addition to the classical analysis of the expression profiles. By using SNVs, CCLONE can identify patterns missed by other methods based only CNVs, MVs or amplified SNVs. This is nicely exemplified by patient P2, where the cellular mosaicism was missed in the previous study (Velten *et al.* 2021), even though the same clones were identified. Another example is patient P3, where our approach finds a different cancer subclone that was overlooked in previous analysis.

Per default, CCLONE takes all variants called from scRNA-seq data as input, and automatically tries to find the most informative variant subset. This approach avoids more complicated and costly variant filtering procedures as used in other methods (Dou *et al.* 2023, Muyas *et al.* 2023) and makes the method easy to apply on new samples. The patterns of occurrence of noninformative variants (s.a. sequencing errors) are expected to be random and will be ignored through the intrinsic capacity of NMF models to deal with random noise. Nevertheless, the set of variants used as input to the wNMF will influence its output. Including too many nonsomatic variants causes a reduction of the signal-to-noise ratio, and might result in losing the clear separation of clones. On the other hand a too strict filtering in which we exclude all uncertain variants can result in the exclusion of somatic events and loss of signal, as shown on the unreliable reference for patient P3 and P4. Another issue can arise from the presence of correlated nonsomatic variants such as cell type specific RNA edits. Here the wNMF would cluster according to these variants and the resulting clones will not reflect genetic information. This highlights the importance of sensible filtering criteria of the variants subset used as input. In the absence of prior knowledge (such as identified through panel testing of known disease genes or WE data), in the ‘Evaluation and selection of results’ step of the CCLONE workflow (see Methods), we try different filtering thresholds for the variants used as input to the wNMF and afterwards determine the best result based on the orthogonality score among the identified clones (Equation 3). This approach [in contrast to including only variants of certain somatic origin (Dou *et al.* 2024, Muyas *et al.* 2024)], allows the model to make use of potential germline variants if they are informative and can result in the capture of CNVs, as was the case for patient A1.

The wNMF step of CCLONE tries to find groups of co-occurring variants across cells in an unsupervised manner. A similar concept has been previously used for demultiplexing scRNA-seq data containing mixed patient samples based on likely germline variants (Huang *et al.* 2019, Heaton *et al.* 2020). The similarity is also highlighted by patient P2, in which we are likely really demultiplexing a sample with mixed genotype. However, demultiplexing different patients is generally a simpler task than identifying subclones in the same patient, mainly because of the higher number of distinctive variants. We compared the wNMF to vireo (Huang *et al.* 2019) on the same input data for all patients analysed in this work. We found the wNMF to perform as good as or better than vireo despite its simplicity compared to the Bayesian model used in vireo (Supplementary Fig. S19).

CCLONE relies on finding groups of variants that co-occur within genetic clones (i.e. the healthy or cancer populations). Therefore, the success of the method depends on capturing sufficiently co-occurring somatic variants in the data. This capture rate depends both on the mean sequencing depth and also on the sequencing technologies. As shown in Section 3.3, 3′ biased technologies such as 10X will miss more variants than technologies with coverage over the full length of the transcript. For cancer samples with low mutational load, such as is expected for most AML patients, we recommend higher sequencing depth (≈1e6) and coverage over the full length of the transcript.

The smaller the group of co-occurring variants and the smaller the clonal cell populations in the input data, the smaller the decrease in model error *E*. One consequence of this, is that very small clones can be missed by the wNMF, as the decrease in *E* approaches background noise. To ensure that both the healthy and cancer populations have sufficient size to be captured by the wNMF, we restrict our analysis to patients that have both sufficient healthy cell types. We recommend each population to be present at frequencies of at least 10% when running CCLONE on new data. Alternatively, prior knowledge during variant selection can also help enhance the signal-to-noise ratio to allow for the identification of smaller clones. In this work, we further use these cell types to annotate and validate the factors, highlighting again the need for sufficient cells of these cell types in the sample. Other prior knowledge on the analysed samples (such as known somatic events present in the data) could be used instead in the absence of this information.

Cancer is continuously evolving through acquisition of new somatic mutations and clonal expansion. Therefore, there is an inherent uncertainty in any attempt to group the cells into separate groups of healthy and cancer cells. Depending on the time of acquisition of each somatic variant, these variants might be present in all, or in different subsets of the cancer cells. This imbalance between variants found in different subclones might be particularly pronounced if the cancer acquires a mutator phenotype, resulting in a higher mutation rate in the corresponding clone (Hanahan and Weinberg 2011). In the presence of multiple subclones with different somatic variants, the subset of variants used as input to the wNMF will then determine which of these we identify. As a result, for early cancer clones, the wNMF is not guaranteed to identify all cancer cells. This is exemplified by patients P1, P3 and A6, where we identify a subset of all cancer cells as the cancer population (Fig. 2C and Supplementary Fig. S4). For patients P1 and P3, we could show that the identified subset of cells corresponds to a genetically distinct population of cells, indicating that we are capturing a subclone of the cancer population.

CCLONE’s success rate (17/32 of all patients analysed in this study and 4/4 of the Smart-seq2 patients data) in using SNVs seen in scRNA-seq data for identification of clonal structure in cancer samples motivates further computational developments in this direction. Our approaches, that requires no previous knowledge about existing mutations, can provide clonal insight into existing datasets without need for additional experiments (such as WE sequencing). In the future, one could consider other probabilistic approaches of handling uncertainty in the variant call data. These could include efficient ways of incorporating prior knowledge (e.g. through known somatic events) into semi-supervised classification algorithms. In this work we focused on extracting the information present in the uncertain SNVs and showing that this layer contains usable clonal information. Future methods could also combine the different information layers provided from nuclear SNVs, CNVs, and MVs. New approaches for joint analysis of the clonal and gene expression information from the same single cells could help us get a more complete picture of the mutational journey of healthy cells towards cancerous states.

## Supplementary Material

btae512_Supplementary_Data

## Data Availability

The raw sequencing data of the two AML datasets are available at the European Genome-Phenome Archive with the accession IDs EGAS00001003414 (Velten *et al.* 2021) and EGAS00001007078 (Beneyto-Calabuig *et al.* 2023). All notebooks necessary to reproduce the results reported in this paper and the anonymized variant call data are available on our GitHub page: github.com/HaghverdiLab/CCLONE_notebooks. A python package of the CCLONE pipeline can be found at github.com/HaghverdiLab/CCLONE.
