# Supplementary material for: Identifying cancer cells from calling single-nucleotide variants in scRNA-seq data
Source: Bioinformatics. 2024 Aug 20;40(9):btae512. doi: 10.1093/bioinformatics/btae512 (PMC11379463; doi:10.1093/bioinformatics/btae512)
Supplement: btae512_Supplementary_Data [file btae512_supplementary_data.pdf]

Supplementary notes and figures for
"Identifying cancer cells from calling single-nucleotide variants in scRNA-seq data"

Valérie Marot-Lassauzaie[1,2], Sergi Beneyto-Calabuig[3,4], Benedikt Obermayer[5], Lars Velten[3,4], Dieter Beule[5,6], Laleh Haghverdi[1,*]

1 Berlin Institute for Medical Systems Biology, Max Delbrück Center (BIMSB-MDC) in the Helmholtz Association, Berlin, Germany
2 Charité – Universitätsmedizin Berlin, corporate member of Freie Universität Berlin and Humboldt-Universität zu Berlin, Berlin, Germany
3 Centre for Genomic Regulation (CRG), The Barcelona Institute of Science and Technology, Dr. Aiguader 88, Barcelona 08003, Spain
4 Universitat Pompeu Fabra (UPF), Barcelona, Spain
5 Berlin Institute of Health at Charité – Universitätsmedizin Berlin, Core Unit Bioinformatics, 10117 Berlin, Germany
6 Max-Delbrück-Center for Molecular Medicine in the Helmholtz Association (MDC), Berlin, Germany

* Laleh.Haghverdi@mdc-berlin.de

Contents

1 Supplementary notes **1**
Note A: Weight matrix W 1
Note B: Select variant subset and number of factors 2
Note C: Label latent factors 2
Note D: Alternatives approaches to label latent factors 3
Note E: Preprocessing of the CRC and lung adenocarcinoma datasets 3

2 Supplementary figures **5**

1 Supplementary notes

Note A: Weight matrix W

As described in the main text, the values in the observation matrix M used as input for the wNMF represent the discretised VAF, while the values in the weight matrix W should reflect the confidence that we have in each value of M.

As most variants observed in the data are not somatic, and we only observe positions with two alleles present, our null model is that the variants are heterozygous and unchanged across cells. Only strong evidence of the contrary should be used by the model. To get an estimation of the confidence in the observed VAF, we extract the observed VAF of known germline heterozygous variants (with expected VAF of 0.5, and unchanged across cells) in each dataset.

In the Smart-Seq2 data, the mean VAF of these variants across cells is close to the expected value of 0.5, but the VAF of individual cells is nearly always 0 or 1 (see Fig S3). This pattern is observed irrespective of the depth of coverage of the position. In other words, we almost always only sample one allele at a time in each cell. Consequently, observing exclusively reference (or exclusively alternative) reads only tells us that this allele is present in that cell. We likely still have one unobserved allele, and set the weight in W to 0.5 to reflect this. Observing both reference and alternative read in the same cell indicates that we observed both alleles, and that observation is thus given a weight of 1. We call this weight model "non-UMI weight model" in Fig S4.

In the 10X data, the observed VAF is close to the expected VAF under a Bernoulli sampling model in which we randomly sample reads for each allele (Fig S3). This is likely due to the presence of

UMIs in the data, allowing us to account for PCR replicates. Given X_{ij}, the coverage in cell i at the variant position j, the probability of missing an allele at the observed coverage is equivalent to failing the Bernoulli process X_{ij} times, with $p = 0.5$: $\binom{X_{ij}}{0}0.5^0 0.5^{X_{ij}} = 0.5^{X_{ij}}$. Here as well, observing both alleles would be given a weight of 1. We call this weight model "UMI weight model" in Fig S4. However, for a practical range of coverage values, the weights between these two models are closely correlated to each other (Fig S4). In fact they are also closely correlated to the weights of a binary model, in which we assign a weight of 0 to positions that are not covered and weights of 1 otherwise (Fig S4). In the Smart-Seq2 model, observing a VAF of $M_{i,j} = 0.5$ is very rare, and consequently most values in the weight matrix will be 0.5. For the 10X model, as the coverage increases, the values in W will also approach one. Based on this the main contributing factor of the weights to the wNMF is to ensure that missing values do not contribute to the cost. Given the small difference between the non-UMI and the UMI weight designs, we went for a simple unified weighting model for both platforms, that considers non-observation of variant or reference as uncertain by assigning a weight of 0.5.

Note B: Select variant subset and number of factors

For every variant subset $V_\circ$, the wNMF takes as input the observation matrix M, the weight matrix W and the number of latent factors K.

As explained in the main text, if the variant set used as input for the wNMF contains too many non-somatic variants, the signal can get lost. The cancer population size, and type of somatic events found (deletion of germline SNV, acquisition of somatic SNV) can greatly vary between samples. Because of this, the ideal variant filtering thresholds can also vary between patients. To account for this, we try different thresholds and run the wNMF on each subset. Per default we try both to include and exclude known germline SNVs (based on common dbSNP variants [1]), and to vary the minimal MAF across cells between 3, 5, 10 and 15.

In the next step, the wNMF identifies patterns of variants with correlated VAF patterns across cell groups (for example a set of variants that is seen only in the cancer population). These variants and cells can be summarised in a factor, resulting in a reduction of E proportional to the number of variants and cells expressing these variants in each group. The higher the number of co-ocurring variants, the higher the drop in E, which results in the first factors capturing the main axes of variant variation in the data. Once all groups of co-occurring variants are found, the remaining factors will capture smaller patterns, until the factors describe only individual variants. Ideally, the inputed number of latent factors K would reflect the number of clones clearly identifyable in the data. While in theory, the number of clones present in the sample should be well-defined, in practice, the number of clones that can be identified will depend on the captured variants. To ensure that the factors are not capturing background noise, we would like K to reflect the number of co-occurring variant groups clearly identifiable from the data. If this information is known, the corresponding K can be used as input to the wNMF. In the absence of prior knowledge, we run the wNMF for a range of K (default of 1 to 5), and try to select the "best" K.

For a range of number of latent factors $\kappa = \{K_0 = 2, \ldots, K_3 = 5\}$, and a set of variant subsets $\nu = \{V_0, \ldots, V_n\}$ with corresponding fitted wNMF cell factor matrices $\{C_{(K_0,V_0)}, \ldots, C_{(K_3,V_n)}\}$ of shape (n_{obs}, κ), we select the best result $C_\star$ as the one that maximises the orthogonality score s between the clones as defined in main text Equation 4.

s as a function of K and the variant subset are shown in figure S1 for all patients analysed in this work.

Note C: Label latent factors

As output of CCLONE, we get the cell factor weights C (n_{obs}, K) and the variant factor weights V (K, n_{vars}). If the method succeeds we expect the factors to reflect genetic clones, i.e. one or multiple factors for healthy clone(s) and one or multiple factors for cancer clone(s). However, the wNMF does not directly label these factors as healthy or cancer.

In this work, we label the factors based on prior knowledge of cancer cell populations. For the AML samples, we know that the cancer does not give rise to either T and NK cells, and use those for

labelling the clones. Clones depleted in these populations ($< 10\%$ of T / NK cells in that clone) are labelled as cancer clones, and clones containing these cell types are labelled as healthy. For patients with too few T/ NK cells (AML Smart-Seq2 patients P2 and P4), we use cancer cell types to label the clones. Clones depleted in Blasts ($< 10\%$) are labelled as healthy, and the others as cancer. For the lung adenocarcinoma and CRC dataset, clones depleted in the "Tumor" (or "Tumour" for CRC) cell type are labelled as healthy clones, and the others as cancer.

We also use these labels to validate the clones. The wNMF tries to find groups of co-occurring variants across cells in an unsupervised manner. However, it is not guaranteed that such groups of variants are present in the data. Another problem could arise if the data contains co-occurring variants of non-somatic origin (for example missed RNA edits, or correlated artefacts). The sum of squared errors E (main text Equation 1), reflects how well the present variation is captured by the wNMF. The orthogonality score s (main text Equation 4), reflects how clear the separation between the cell factors is in the data, and thus the expected signal-to-noise ratio. Neither s, nor E inform us whether the captured variation reflects genetic clones. To ensure that the variation captured by the wNMF corresponds to separation between healthy and cancer, we use instead the known cell types to validate the factors. If no factors can be labelled as either healthy or cancer (i.e. all factors contain healthy or cancer cell types), we assume that the model has failed.

Note D: Alternatives approaches to label latent factors

In the absence of reference cell states (i.e. all cell states are mixture of healthy and cancer), prior knowledge on the variants used as input to the wNMF could be used for labelling and validation instead. If some variants correspond to known somatic events we could then verify that these have different weights between the factors, and use these for labelling of the factors. Another alternative would be to carefully curate the variant set used as input to the wNMF and ensure that only likely somatic events are used. By excluding all other potential sources of co-occurring variants, we would then ensure that the identified variation corresponds to somatic variation. However this exclusion of uncertain variants is potentially time consuming and error prone. It could also come at the cost of missing resolution for several lineages if they contain no well-covered curated variants.

In the absence of prior knowledge on the variants, a more careful analysis of the variants enriched in each factor could help in understanding, labelling and validating the factors. If we find multiple neighbouring variants found at VAF≈ 0.5 in one population and either lost of fixated in the other population, they could point towards a deletion or LOH in that region (as shown for patient A1 in Figure 3, or P3 in Figure S3). Another option would be to look for potential driver variants in the enriched variants. This could be done by testing whether these variants are predicted to have an effect and are found within disease-associated genes. As shown in this work, the patterns of enriched variants can be very different between patients. Consequently, this approach would have to allow for flexibility and evaluating the enriched variants might be time consuming. It would also come at the cost of excluding patients with no identifiable somatic events in the enriched set.

Note E: Preprocessing of the CRC and lung adenocarcinoma datasets

The reference cancer cell labels in the CRC and lung adenocarcinoma datasets are equivalent to the ones used in the original studies and based on CNVs. The wNMF needs the presence of at least 2 genetic populations at sufficient frequencies to find patterns of variant co-occurrence. Because of this, we exclude patients that have almost only ($>97\%$) tumour cell types, leaving us with 7 patients for the lung adenocarcinoma data (Figure S12A) and 6 for the CRC data (Figure S12B).

2 Supplementary figures

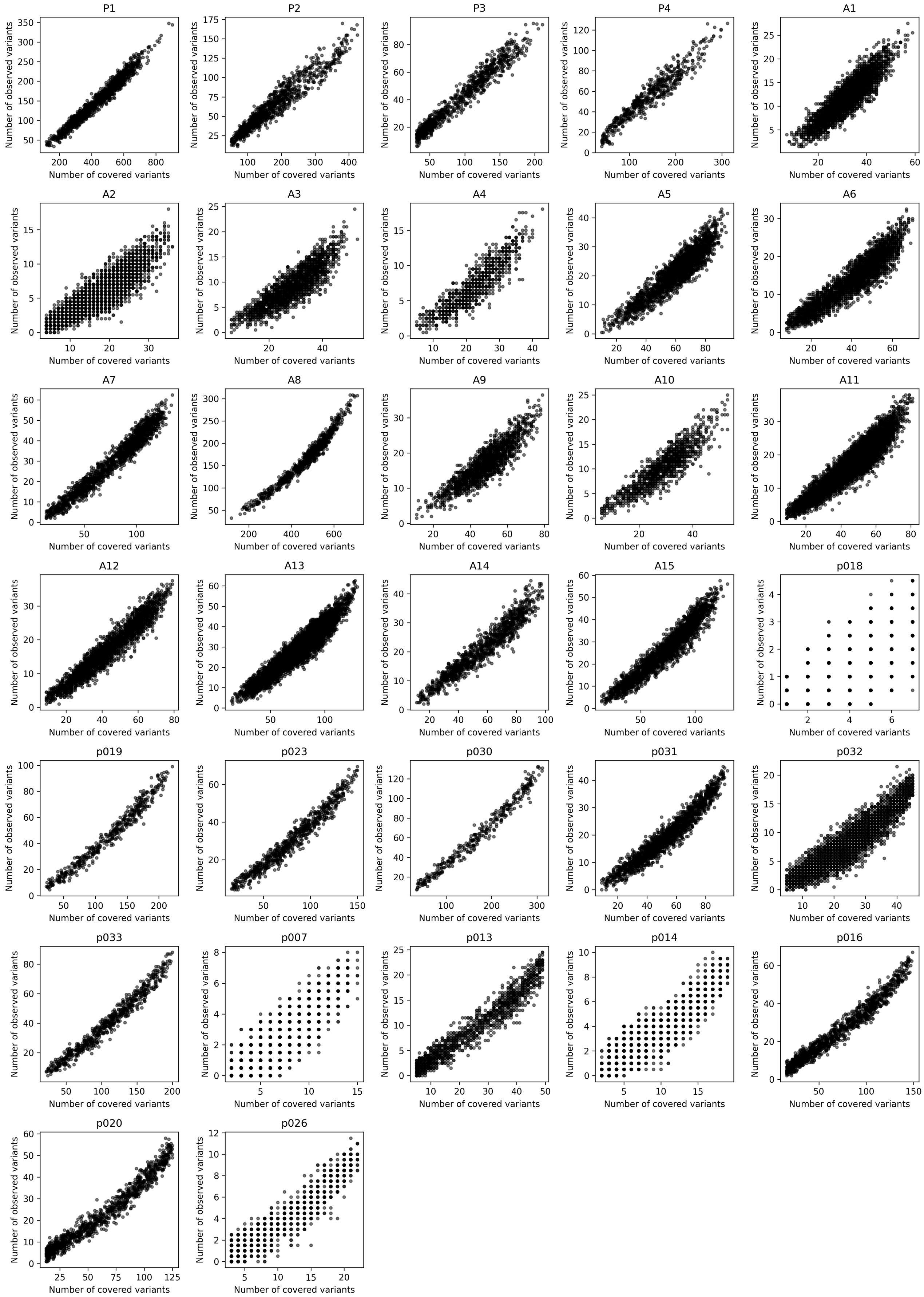

Figure S1: Cell variant as a function of the coverage. We filter the germline variants and RNA edits based on database annotation. We further filter low frequency (<2%) variants, and then show the number of observed variants as a function of the number of covered patients for all patients analysed in this work. Because of the lenient filtering, we still have many nonsomatic variants in the data. Because of this, the overall number of observed variants to identify the cancer cells is directly

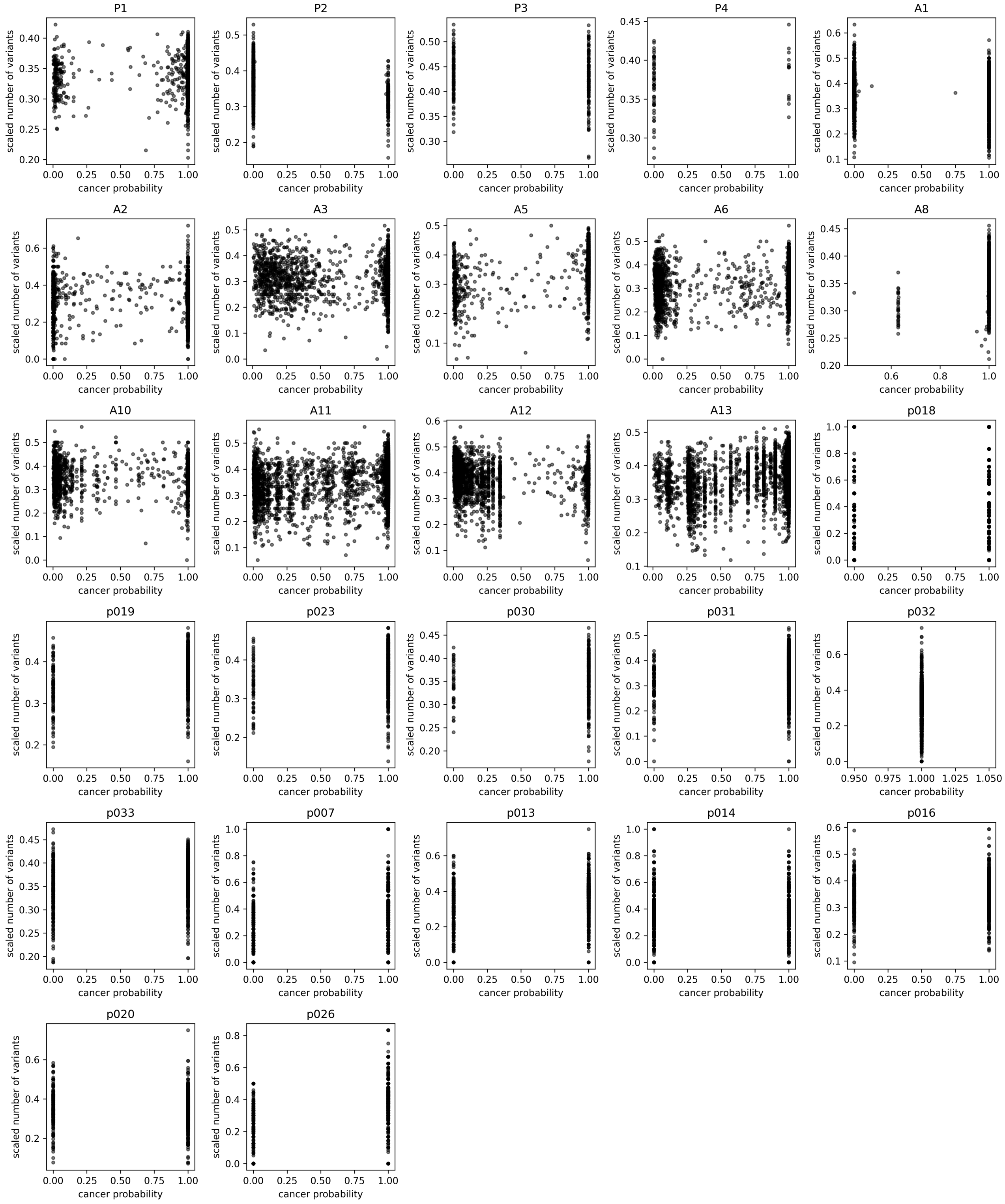

Figure S2: Cell variant as a function of the coverage. We filter the germline variants and RNA edits based on database annotation. We further filter low frequency (<2%) variants, and then show the number of observed variants divided by the number of covered patients as a function of the reference clonal probability for all patients with reference annotation analysed in this work. Because of the lenient filtering, we still have many nonsomatic variants in the data and the scaled number of variants does not correlate to cancer cell status.

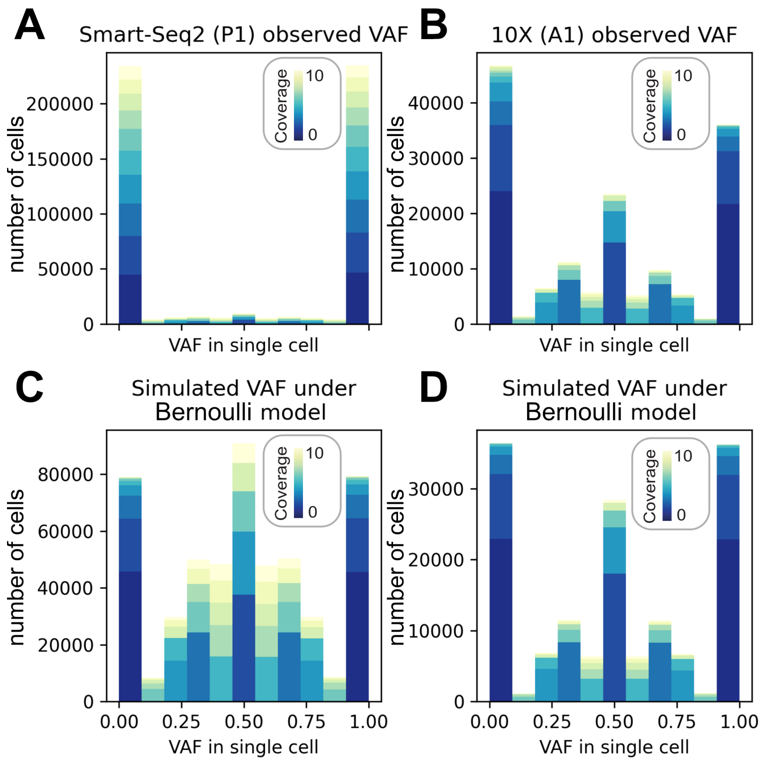

Figure S3: Variant allele frequency of germline heterozygous variants in single cell. We select known heterozygous germline variants and report the observed VAF in single cells in a histogram for a Smart-Seq2 patient (in A) and a 10X patient in (B). Bellow we show the simulated VAF under a Bernoulli model at the same coverage as the observed coverage of the Smart-Seq2 patient P1 in (C) and of the 10 patient A1 in (D). For the Smart-Seq2 data, the observed VAF of individual cells is nearly always 0 or 1, and this pattern is independent of the depth of coverage of the position. For the 10X data, the observed VAF is much closer to the expected VAF under a Bernoulli sampling model.

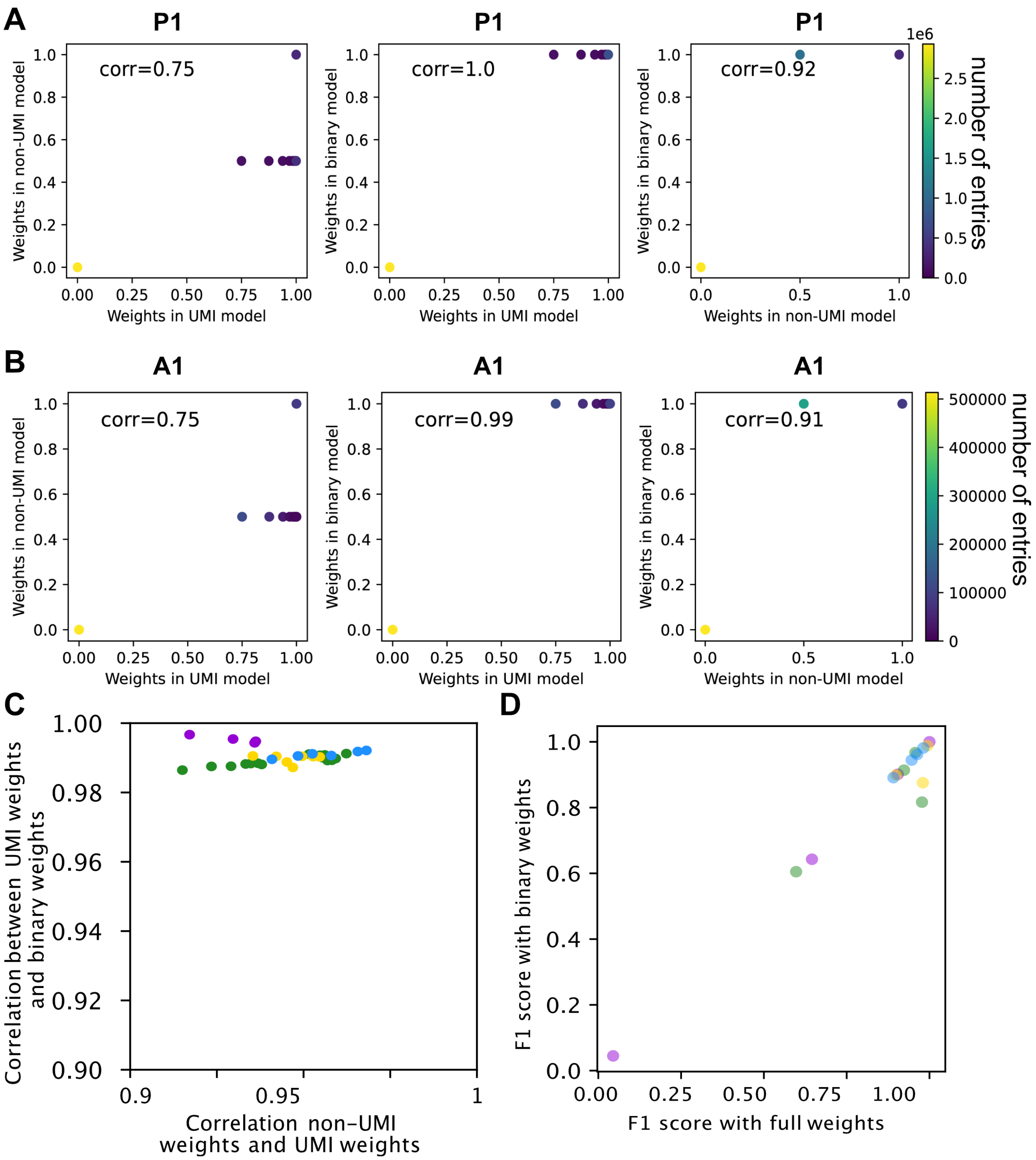

Figure S4: Correlation of weight models. We compare the weights from the UMI model, the non-UMI model and a binary weight model in which we assign a weight of 0 to positions that are not covered and weights of 1 otherwise. Overall, we find a very high degree of agreement between the different weight models. This is shown by comparing the weights of the different models for a Smart-Seq2 patient in (A), and a 10X patient in (B). In (C), we show the correlation of the weights for all patients analysed in this work. Running CCLONE with our weight models and a binary weight model gives us very similar performance as shown in (D), although there is a slight increase in favour of the full weight model for some patients.

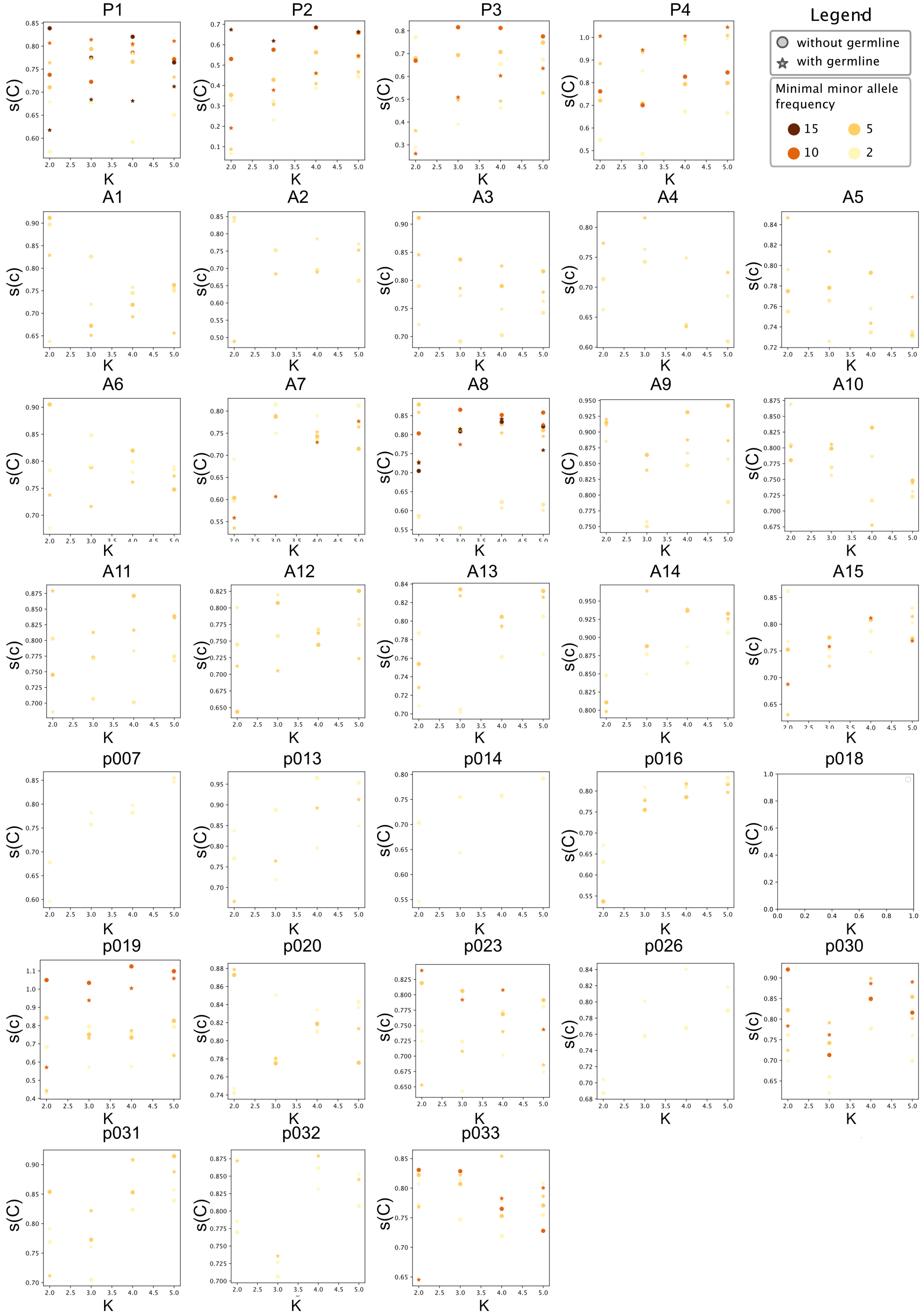

Figure S5: Orthogonality score for real datasets. We show the orthogonality score (main text equation 5) used to determine the number of clones for all patients analysed in this work.

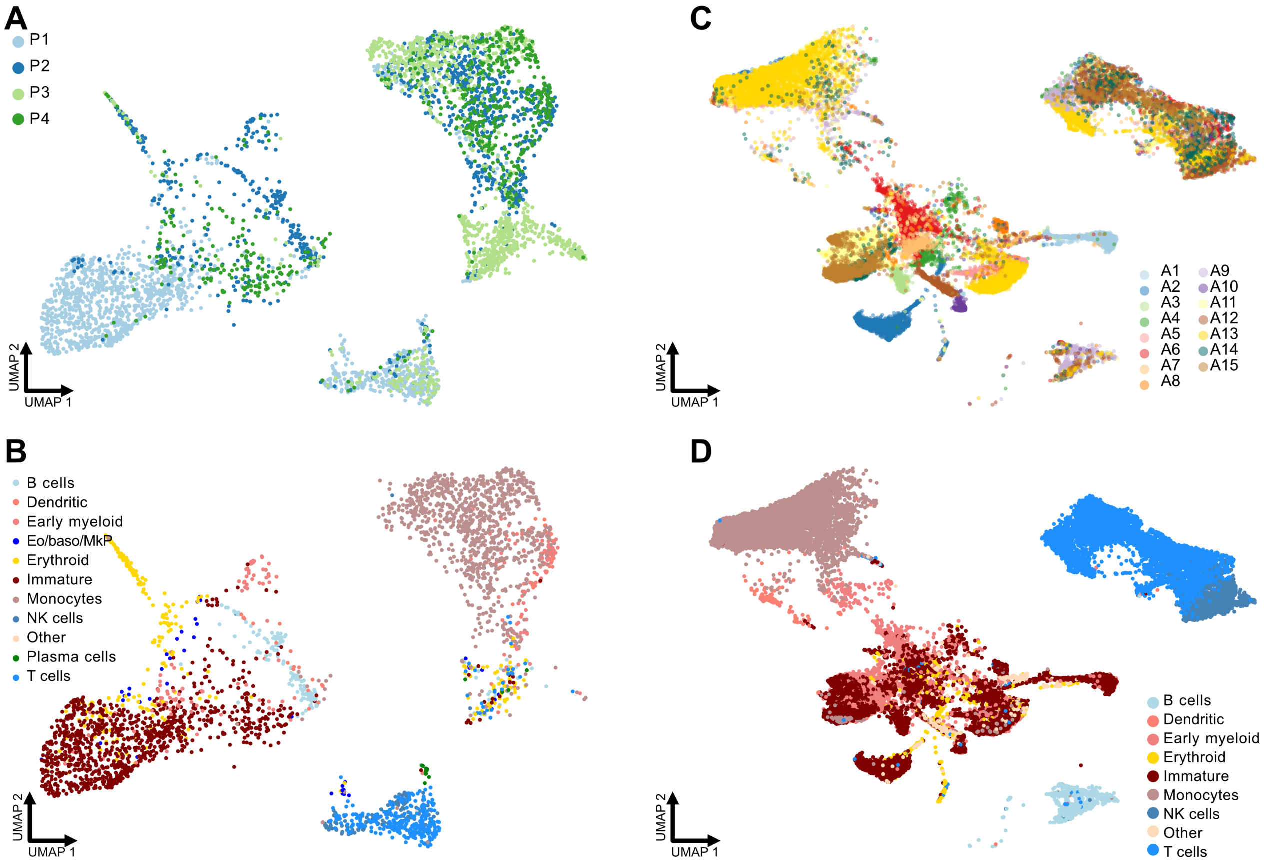

Figure S6: UMAP plots showing the patient labels and detailed cell type labels and for the AML Smart-Seq2 dataset [2] respectively in (A) and (B) and the AML 10X patients [3] in (C) and (D). The UMAP were calculated in the original publications. The cell types shown in (D) are extracted from the original publication. For consistency, the cell types labels in (B) were computed in the same way as in (D) [3]. The cells were projected onto a reference atlas of human hematopoiesis [4] as described in [4].

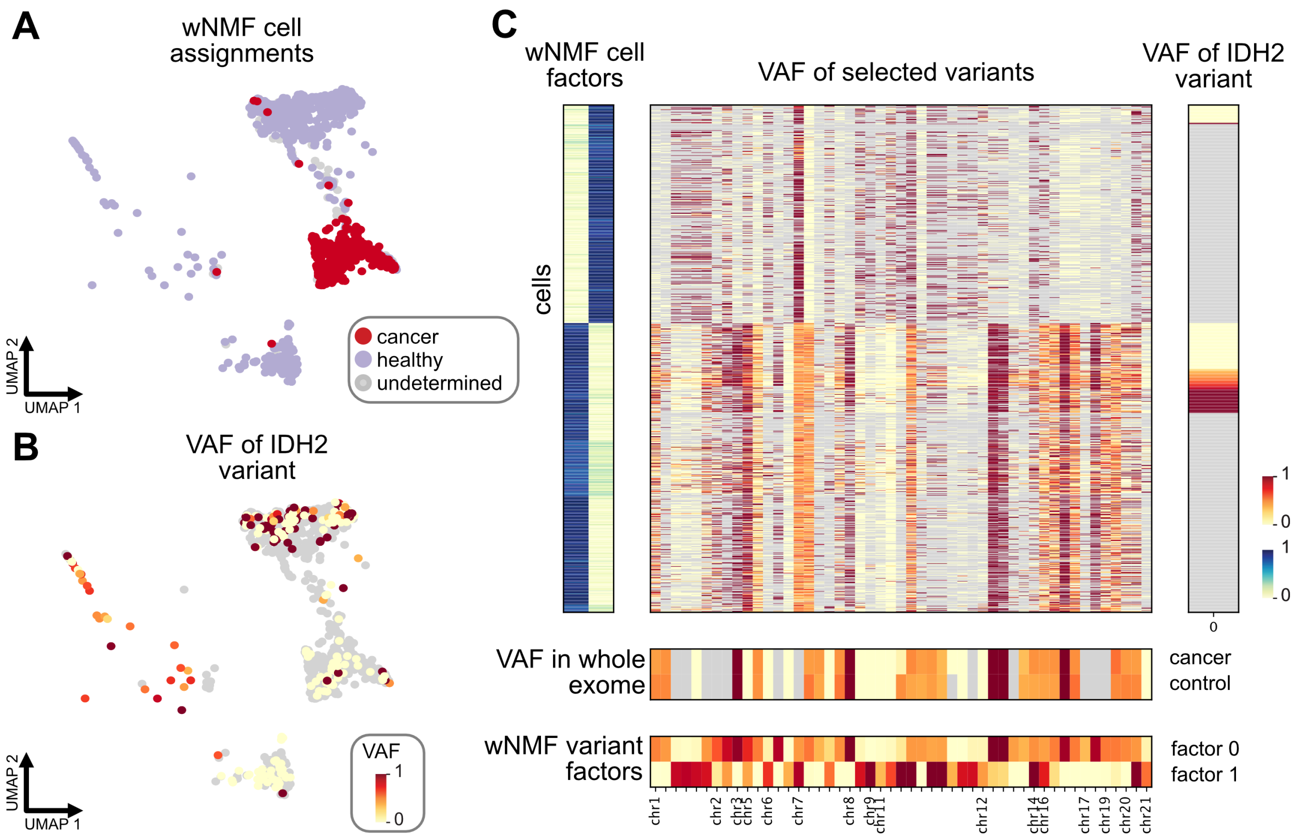

Figure S7: (A) UMAP plot showing our cell labels for patient P3 of the AML Smart-Seq2 dataset [2]. (B) UMAP plot showing the VAF of the cancer driving IDH2 somatic variant used to label the cancer cells in the original publication. Cells in grey have insufficient coverage of that variant ($\leq$2 reads). (C) VAF of selected variants for the cells of patient P3. The cells are sorted by cell factors and the subset of variants are selected based on difference between the factors ($\geq$0.3). Grey values have too low coverage ($\leq$ 2 reads for scRNA and $\leq$ 5 for whole exome data). The right-most heatmap shows the VAF of the IDH2 variant, which is found in a subset of the cells of factor 1.

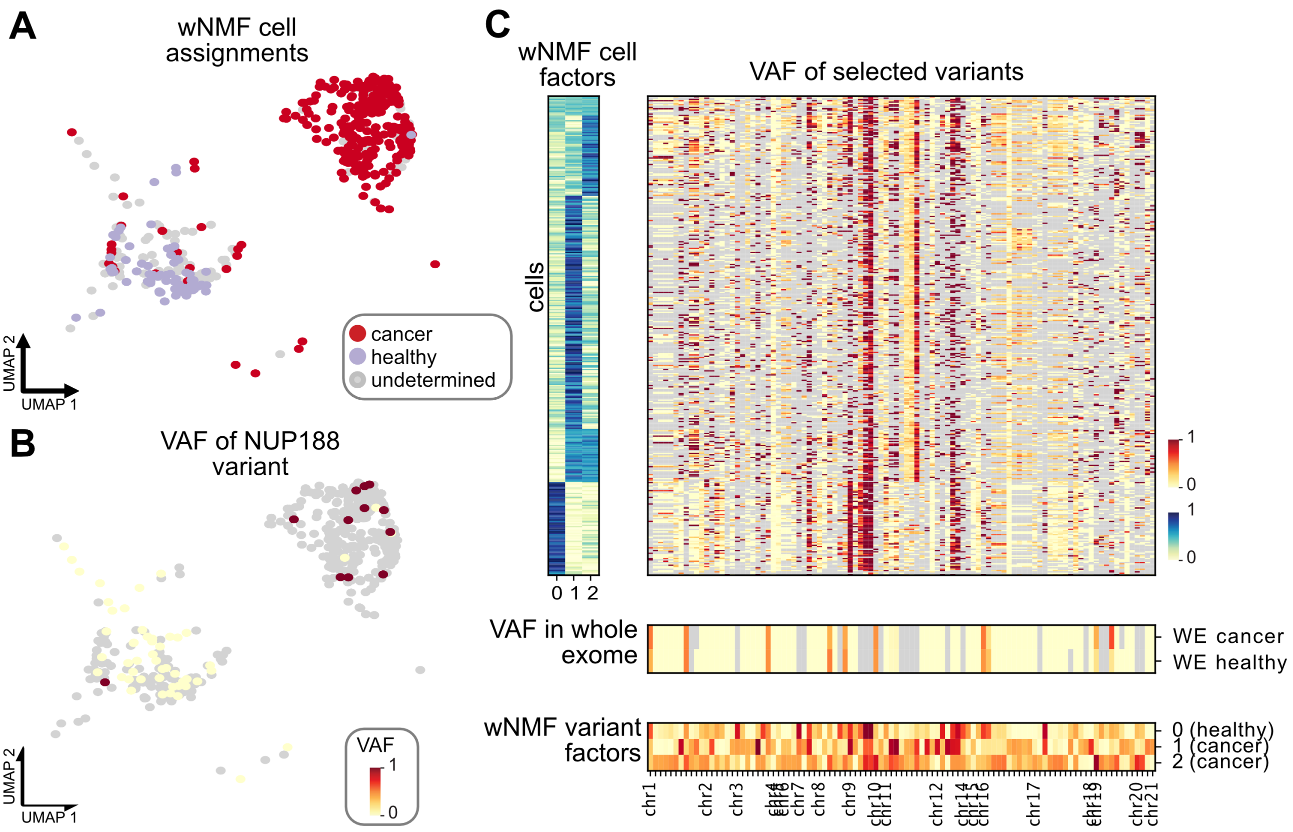

Figure S8: (A) UMAP plot showing our cell labels for patient P4 of the AML Smart-Seq2 dataset [2]. (B) UMAP plot showing the VAF of the cancer driving NUP188 somatic variant used to label the cancer cells in the original publication. Cells in grey have no coverage of that variant. (C) VAF of selected variants for the cells of patient P3. The cells are sorted by cell factors and the subset of variants are selected based on difference between the factors ($\geq$0.3). Grey values have too low coverage ($\leq$ 2 reads for scRNA and $\leq$ 5 for whole exome data).

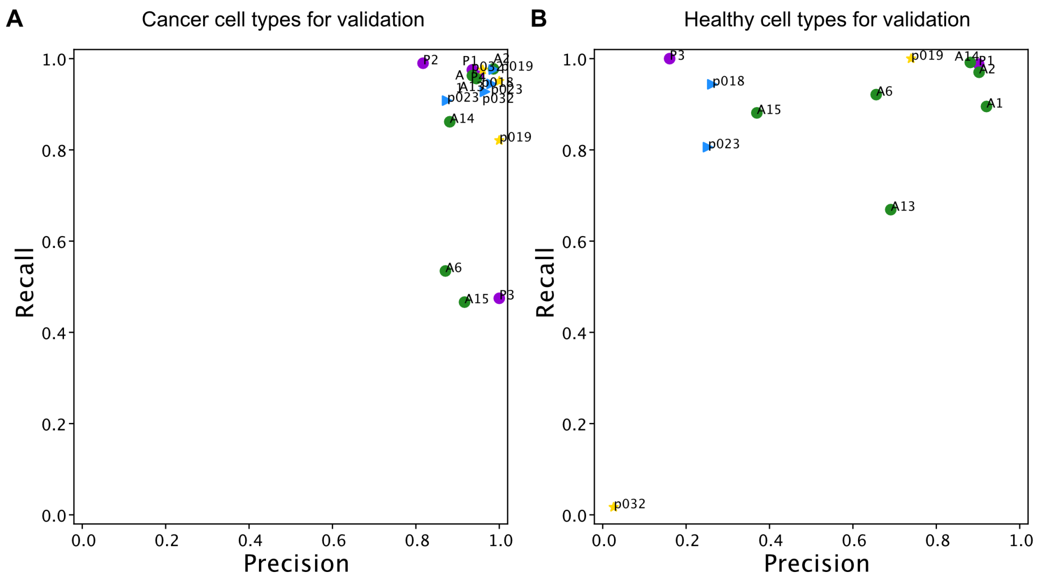

Figure S9: We compare our cancer cell labels to a reference cell labels provided by the cell types. In (A) all Blasts for [2], Early myeloid, Erythroid, Immature or Monocytes for [3] and all Tumor cells for [5] are called cancer and compared to our cancer cell assignments. Patients with lower recall are missing some cancer cells and our labels are potentially catching only a subclone of the full cancer population. In (B), all T/NK cells for [2], B cells, NK cells or T cells for [3] and all Ciliated or Club cells for [5] are called healthy and compared to our healthy cell assignments. We expect more cells than only these cell types to be healthy cells, which explains the lower precision for some patients.

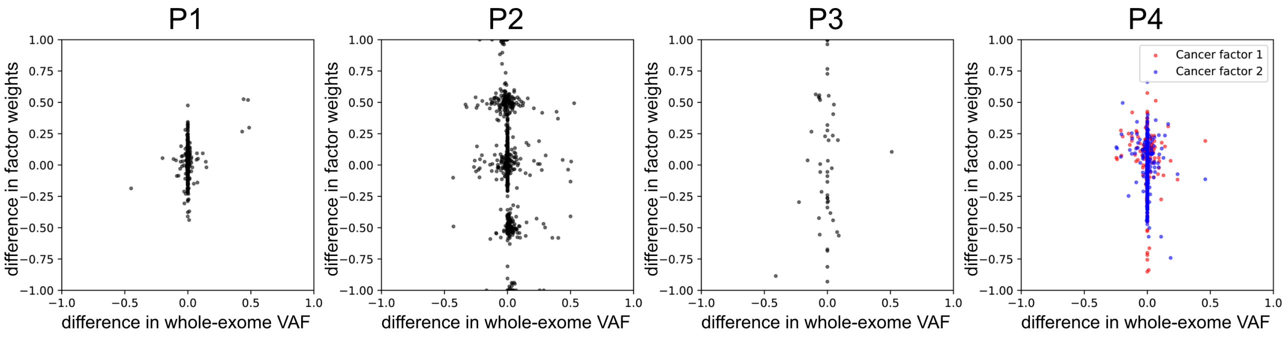

Figure S10: We compare the difference in variant allele frequency between the cancer and healthy whole-exome sample on the x-axis ($VAF_{WE:cancer} - VAF_{WE:healthy}$), to the difference in weight between the cancer and healthy factors on the y-axis ($V_{cancer} - V_{healthy}$). One point represents one variant. We show only variants that have sufficient coverage in all wNMF cell factors (>20% of cells covered) and in the whole-exome data (≥5 reads found both in healthy and cancer). Points enriched in the whole-exome cancer versus healthy will have higher values on the x-axis and points enriched in the cancer factors will have higher values on the y-axis. For patient P4 we found two cancer factors, each shown in one color. We note that for patient P2, as the sample is a mixture of genotypes (patient and donor), and the WE cannot be used for validation of the factors. For P3, we find a cancer subclone that does not contain the only nuclear variant found in the WE cancer (as described in the text).

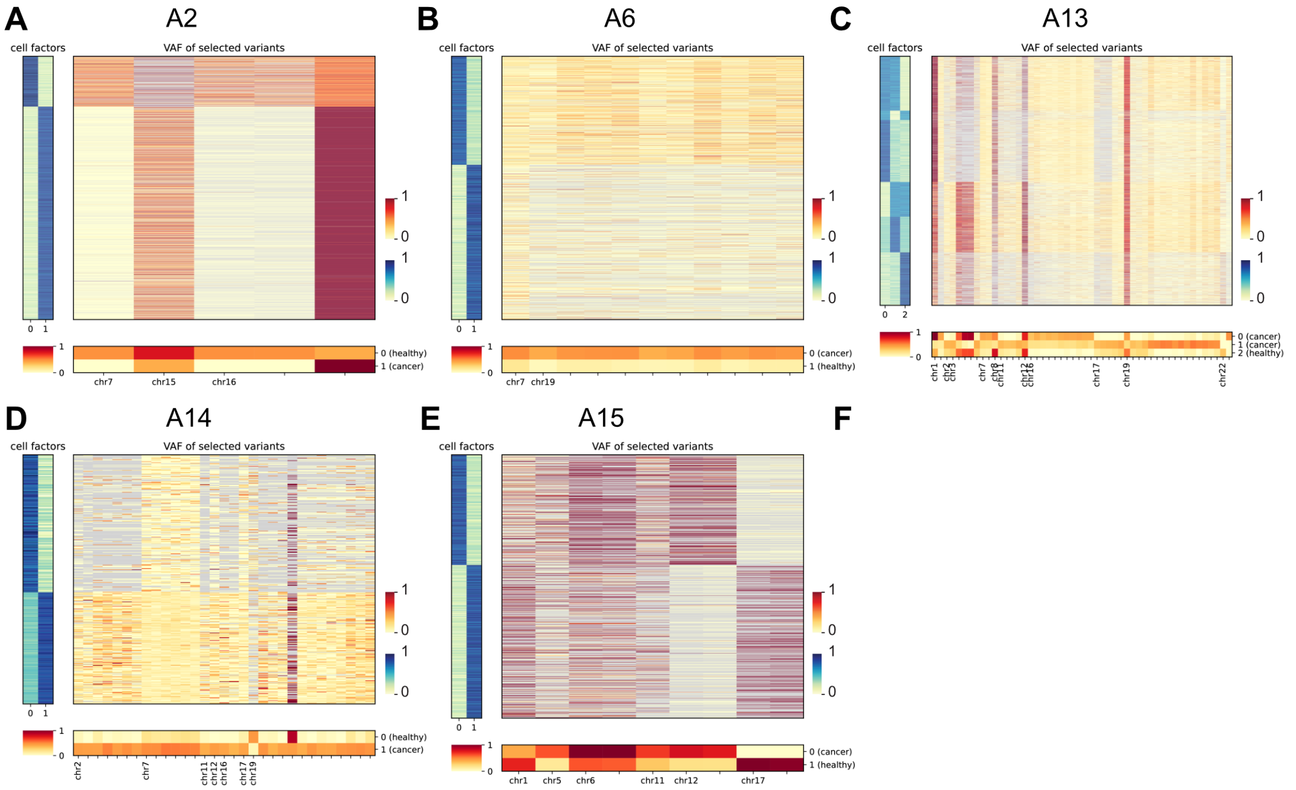

Figure S11: VAF of selected variants for all AML 10X patients where CCLONE succeeds in finding clones [3] (excluding A1 which is shown in main Figure 3B). The cells are sorted by cell factors and the subset of variants are selected based on difference between the factors ($\geq$0.3). Grey values have coverage $\leq$ 2 reads.

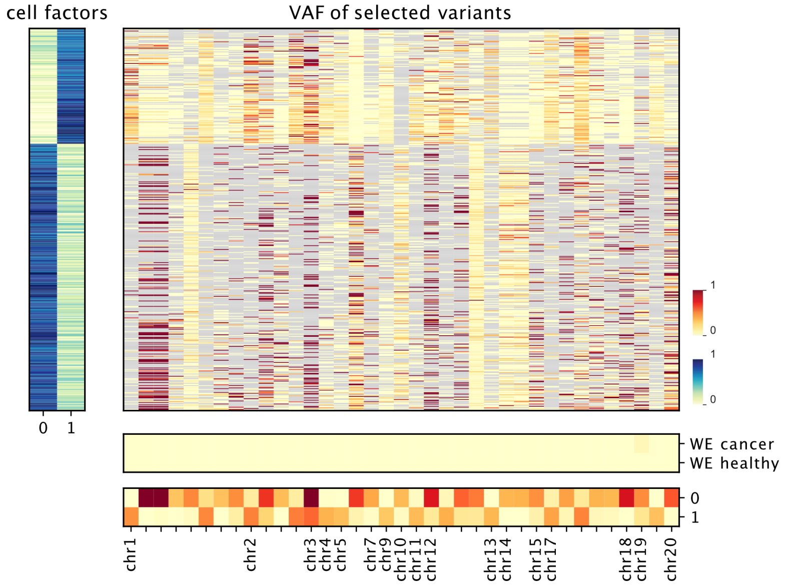

Figure S12: VAF of selected variants for P2 excluding donor cells. The cells are sorted by cell factors and the subset of variants are selected based on difference between the factors ($\geq$0.3). Grey values have coverage $\leq$ 2 reads.

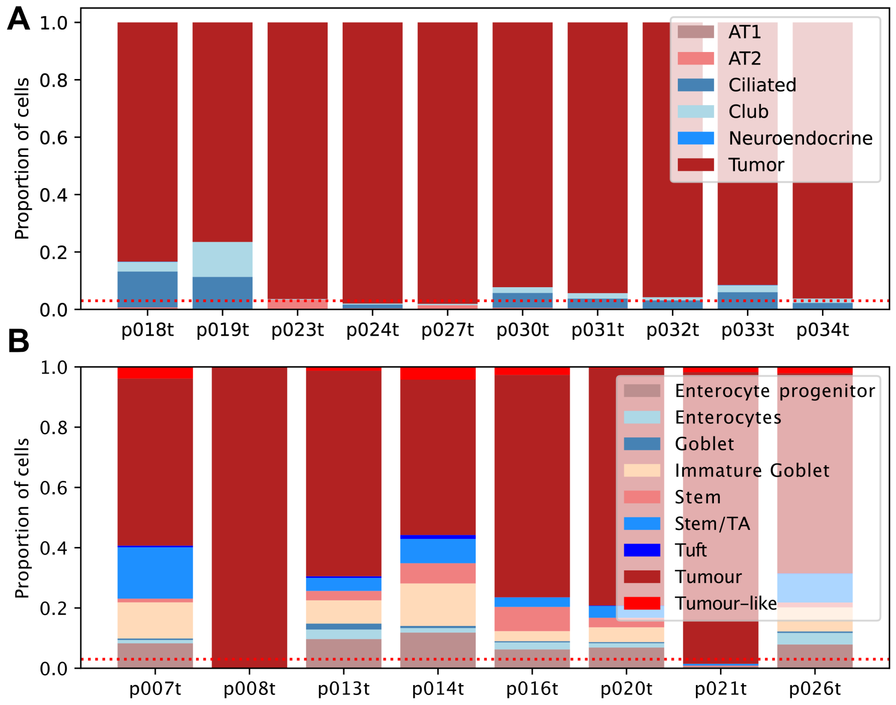

Figure S13: Proportion of cells assigned to each cell type for the tumor sample of each patient of the lung adenocarcinoma data in (A) and of the CRC data in (B). The dotted line shows the threshold used for patient filtering. In (A) p024 and p027 were excluded because they have too few non-tumor cells in the tumor sample. p034 was filtered out due to the very low total number of cells (132 cells). In (B) p008 and p021 were excluded as they had too few non-tumor cells in the sample

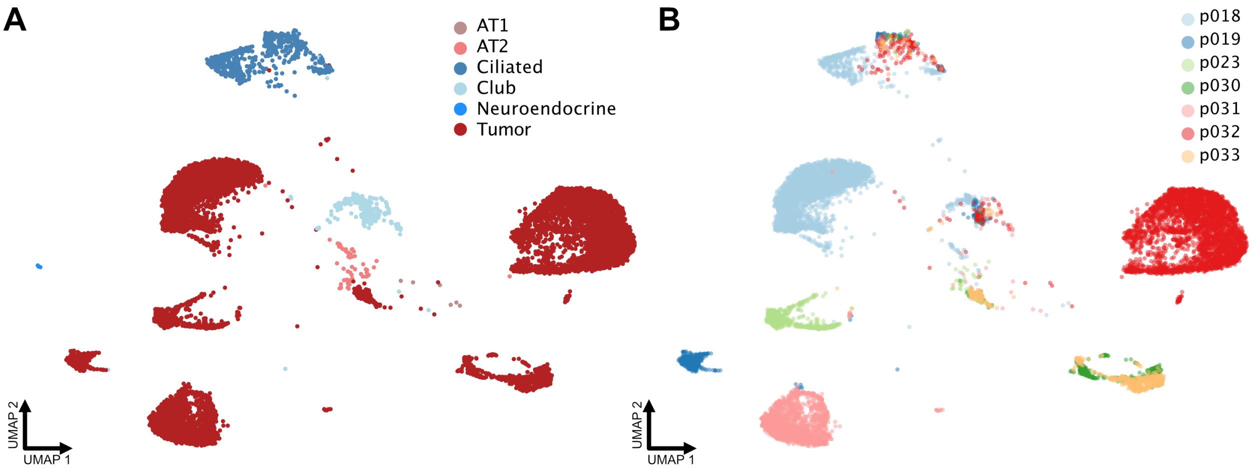

Figure S14: UMAP plots showing the detailed cell type label and patient labels for the lung adenocarcinoma dataset [5] respectively in (A) and (B). The UMAP and cell type labels were calculated in the original publications.

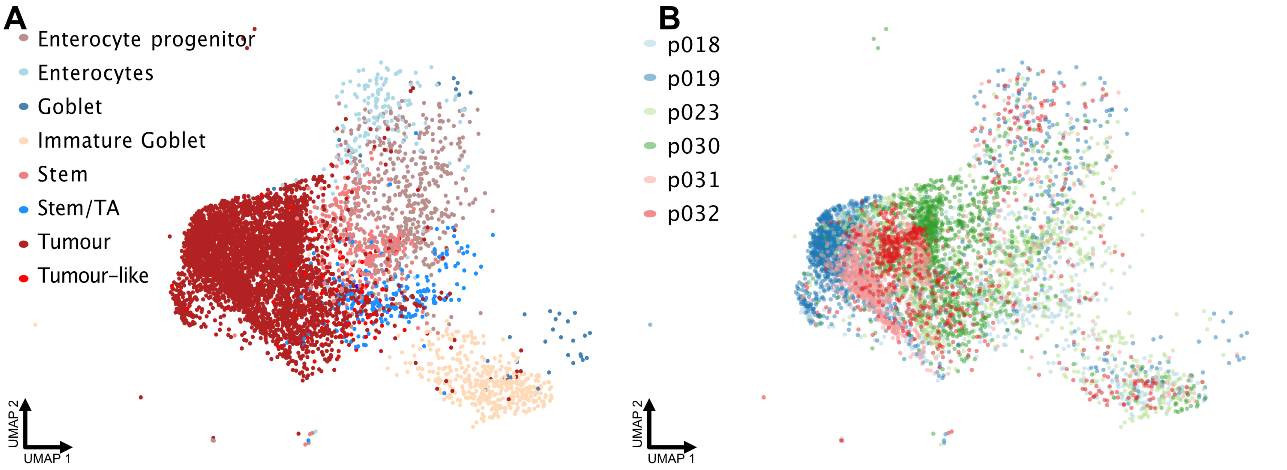

Figure S15: UMAP plots showing the detailed cell type label and patient labels for the CRC dataset [6] respectively in (A) and (B). The UMAP and cell type labels were calculated in the original publications.

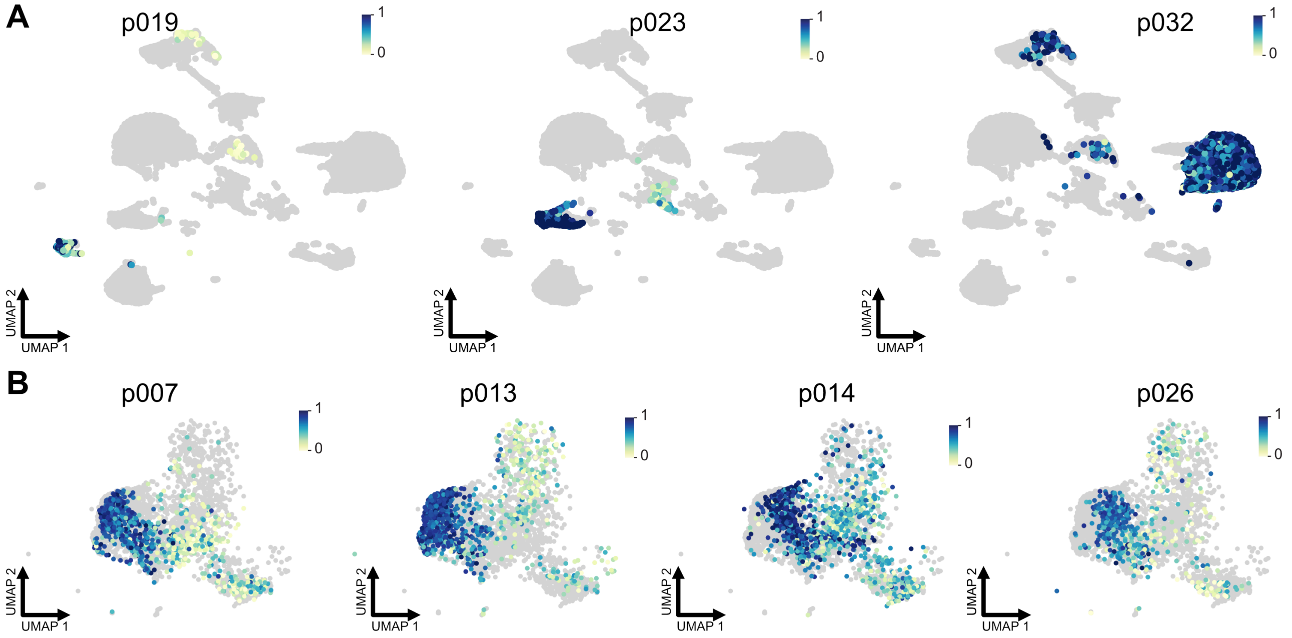

Figure S16: UMAP plots showing the CCLONE cancer cell weights for the lung adenocarcinoma dataset [5] for patients p019, p023 and p033 in (A) and for the CRC data [6] for patients p007, p013, p014 and p026 in (B).

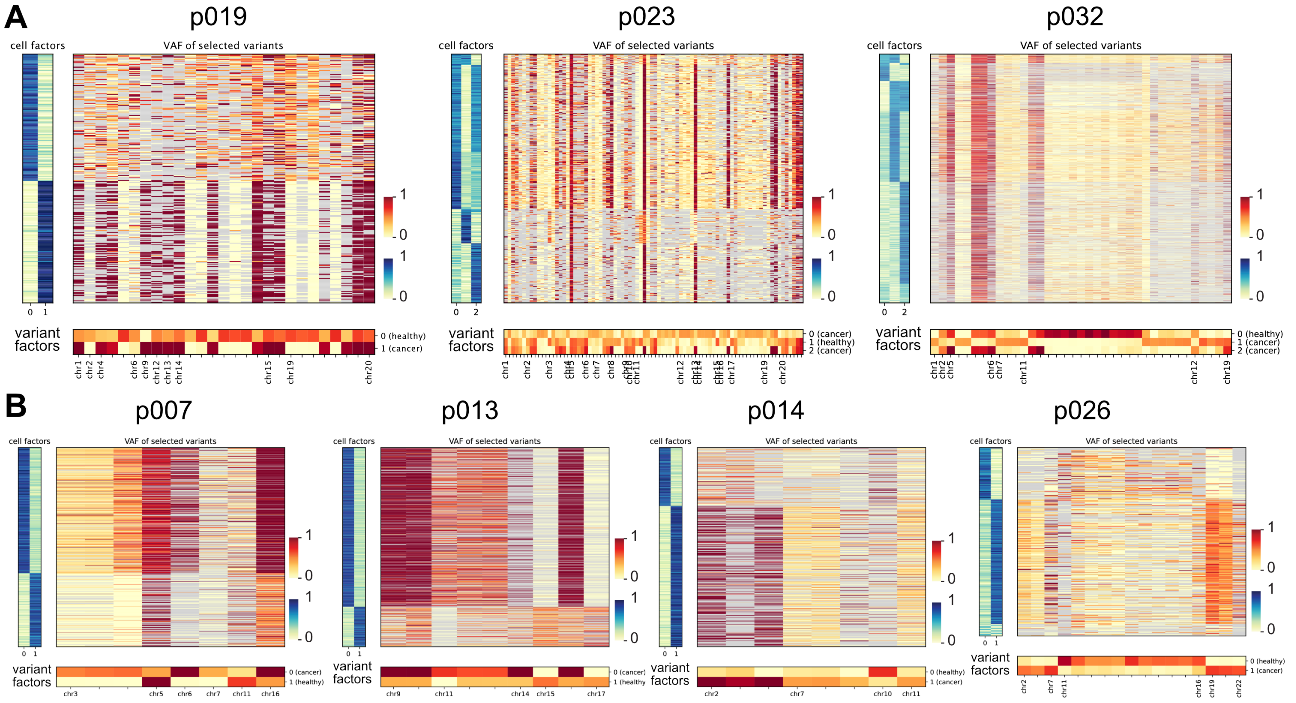

Figure S17: VAF of selected variants for for the lung adenocarcinoma dataset [5] for patients p019, p023 and p033 in (A) and for the CRC data [6] for patients p007, p013, p014 and p026 in (B). The cells are sorted by cell factors and the subset of variants are selected based on difference between the factors (≥0.3). Grey values have coverage ≤ 2 reads.

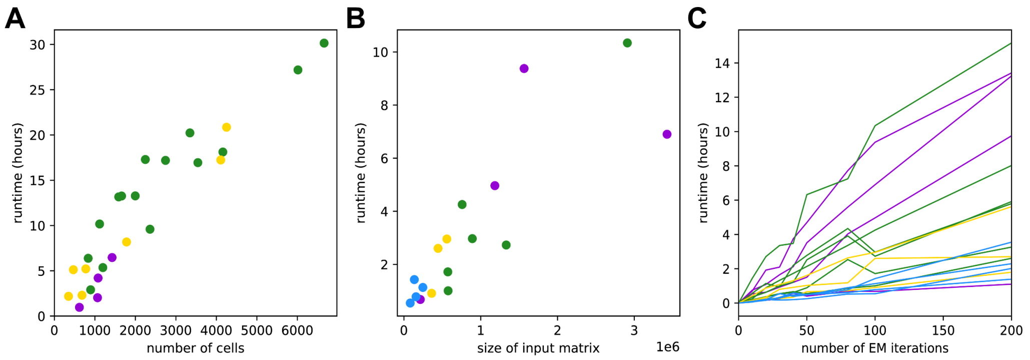

Figure S18: (A) Overview of the runtime for variant calling for chromosome 1 with Cellsnp-lite on a Dual Xeon E5-2650v2 (8cores/2.6GHz) and 15 GB of memory for all patients analysed in this work. The variant calling was performed for all chromosomes in parallel. (B) Runtime of the wNMF with default parameters as a function of the size of the input variant call matrices (number of cells times number of variants) (C) Runtime of the wNMF as a function of the number of EM iterations (default set to 100).

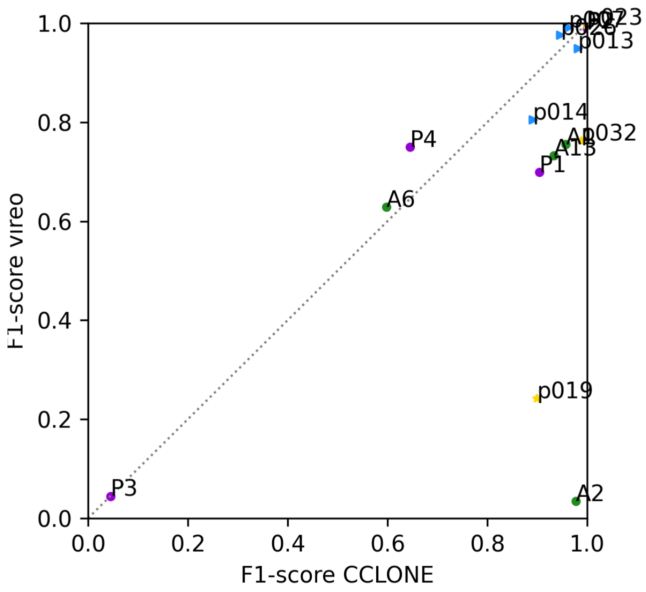

Figure S19: We compare the performance of CCLONE using the wNMF on the x-axis, to the performance of vireo on the same input data on the y-axis. We observe a high agreement between the methods, but better performance for the wNMF in some samples.

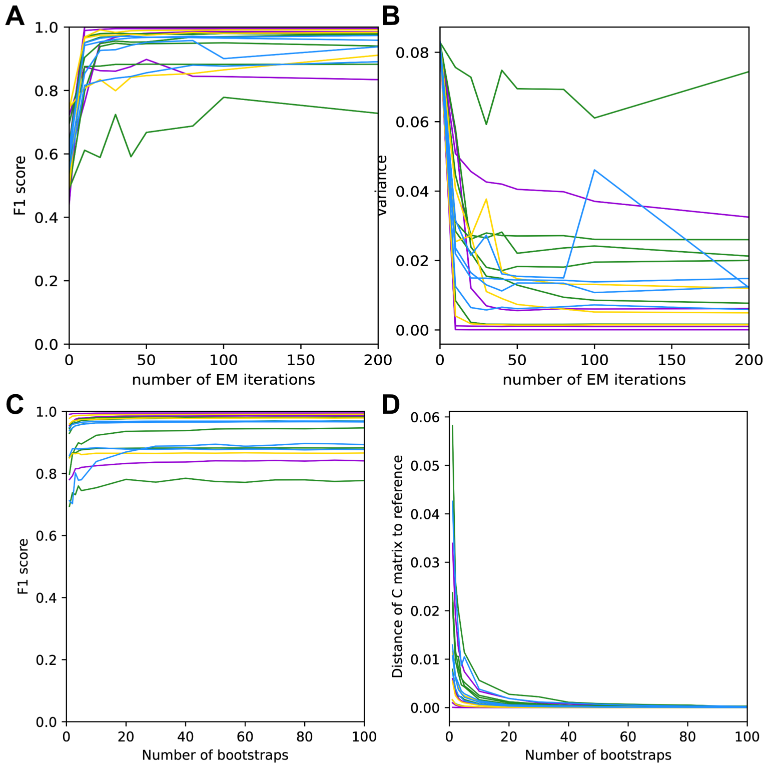

Figure S20: We test the robustness of the solution of the wNMF as a function of the number of EM iterations. We show the F1 score in (A) and the variance of the solutions over multiple runs in (B). We also test the robustness of the solution as a function of the number of bootstraps. We show the F1 score in (C) and the mean distance of the solution to the final one in (D).

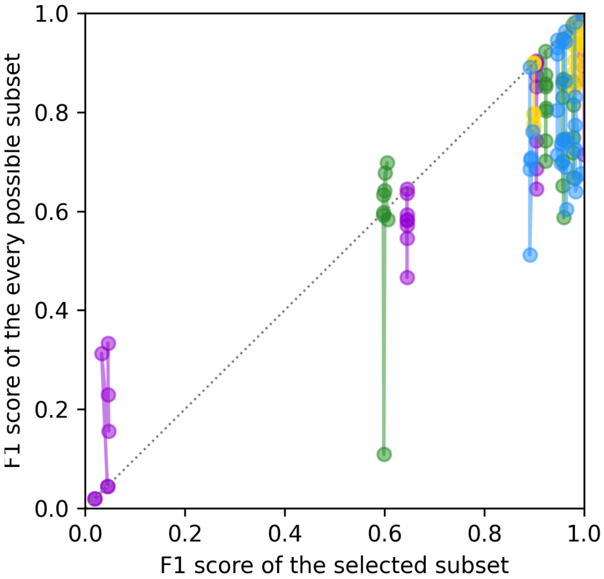

Figure S21: We test the effectiveness of selecting the variant subset based on the orthogonality score of the solution (main text Equation 4). We compare the F1 score of our selected solution to the F1 score of all subsets. Overall we can see that this relatively simple approach is able to select the best subset in nearly all the cases, and this without using any prior knowledge on the data.

References

1. S. T. Sherry et al. dbSNP: the NCBI database of genetic variation. *Nucleic Acids Res*, 29(1):308–311, Jan 2001.

2. Lars Velten et al. Identification of leukemic and pre-leukemic stem cells by clonal tracking from single-cell transcriptomics. *Nature Communications*, 12(1):1366, March 2021.

3. Sergi Beneyto-Calabuig et al. Clonally resolved single-cell multi-omics identifies routes of cellular differentiation in acute myeloid leukemia. *Cell Stem Cell*, 30(5):706–721.e8, May 2023.

4. S. Triana et al. Single-cell proteo-genomic reference maps of the hematopoietic system enable the purification and massive profiling of precisely defined cell states. *Nat Immunol*, 22(12):1577–1589, Dec 2021.

5. P. Bischoff et al. Single-cell RNA sequencing reveals distinct tumor microenvironmental patterns in lung adenocarcinoma. *Oncogene*, 40(50):6748–6758, Dec 2021.

6. F. Uhlitz et al. Mitogen-activated protein kinase activity drives cell trajectories in colorectal cancer. *EMBO Mol Med*, 13(10):e14123, Oct 2021.
